# Comparative theranostic efficacy of ^177^Lu- and ^161^Tb-labeled A1K2 SdAb in mesothelin-positive tumors

**DOI:** 10.1007/s00259-025-07723-z

**Published:** 2025-12-27

**Authors:** Émilien N’Guessan, Florian Raes, Mitra Ahmadi, Sandrine Bacot, Laurent Dumas, Julien Leenhardt, Amaury du Moulinet d’Hardemare, Marlène Debiossat, Clémence André, Jean Boutonnat, Jérôme Durivault, Christopher Montemagno, Jean-Luc Lenormand, Loïc Djaïleb, Ulli Köster, Michiel Van de Voorde, Stijn Ramaekers, Ken Verguts, Catherine Ghezzi, Pascale Perret, Charlotte Lombardi, Alexis Broisat

**Affiliations:** 1 University Grenoble Alpes, INSERM U1039, LRB, Grenoble, 38000 France; 2 Département de Médecine Nucléaire, University Grenoble Alpes, INSERM U1039, CHU Grenoble Alpes, LRB, Grenoble, 38000 France; 3 University Grenoble Alpes, CNRS 5250, DCM, Grenoble, 38000 France; 4 Département de Pathologie, University Grenoble Alpes, CHU Grenoble Alpes, Grenoble, 38000 France; 5 Biomedical Department, Centre Scientifique de Monaco, Monaco, 98000 Principality of Monaco; 6 University Grenoble Alpes, CNRS 5525, TIMC-Tree, Grenoble, 38000 France; 7 Institut Laue-Langevin, Grenoble, 38000 France; 8 SCK CEN, Nuclear Medical Applications, Mol, Belgium

**Keywords:** Radio-ligand therapy, SdAb, Mesothelin, Terbium-161, Lutetium-177, Triple negative breast cancer

## Abstract

**Purpose:**

Mesothelin (MSLN), a 40 kDa glycoprotein normally confined to mesothelial cells, is overexpressed in several malignancies, including triple-negative breast cancer (TNBC). The anti-mesothelin single-domain antibody (sdAb, or “nanobody^®^”) DOTA-A1K2, previously validated for positron emission tomography (PET) imaging using site-specific ^68^Ga radiolabeling, may also serve as a radio-theranostic agent when labeled with ^177^Lu. Moreover, ^161^Tb has recently been proposed as an alternative to ^177^Lu that might provide additional efficacy due to the emission of Auger electrons. The aim of this study was to evaluate *in vitro* and *in vivo* the therapeutic effect of [^177^Lu]Lu-DOTA-A1K2 and [^161^Tb]Tb-DOTA-A1K2.

**Methods:**

Biodistribution was assessed in mice from 1 to 168 h post-injection. Therapeutic efficacy was tested using MDA-MB-231 TNBC cells transfected or not with MSLN. Clonogenic assays were performed after 24 h incubation with either radiotracer. *In vivo*, efficacy was evaluated over 9 weeks after a single intravenous injection of 2, 5, 10, or 20 MBq.

**Results:**

DOTA-A1K2 was successfully radiolabeled with both isotopes with RCP > 95%. *In vitro*, [^161^Tb]Tb-DOTA-A1K2 was significantly more potent than [^177^Lu]Lu-DOTA-A1K2 in inhibiting colony formation (*p* < 0.01). *In vivo* in mice, the radiotracers exhibited similar biodistribution profiles. The administration of 5, 10 or 20 MBq of either [^177^Lu]Lu-DOTA-A1K2 or [^161^Tb]Tb-DOTA-A1K2 inhibited tumor growth compared to controls (*p* < 0.01 vs. vehicle), while no effect was observed at 2 MBq (p = NS). However, no differences in efficacy were observed between the two isotopes at any dose.

**Conclusions:**

This work provides the first sdAb-based theranostic approach targeting mesothelin-positive tumors. *In vivo* in mice, both [^177^Lu]Lu-DOTA-A1K2 and [^161^Tb]Tb-DOTA-A1K2 accumulated in tumors. *In vivo* efficacy was found to be comparable, despite the superior *in vitro* efficacy of ^161^Tb. Further studies are warranted to clarify this discrepancy, which could potentially result from mesothelin shedding that prevents Auger electrons from reaching tumor cells.

**Supplementary Information:**

The online version contains supplementary material available at 10.1007/s00259-025-07723-z.

## Introduction

Mesothelin (MSLN) is a 40 kDa glycoprotein anchored to the cell membrane via a glycosyl-phosphatidylinositol (GPI) linkage. Its expression in healthy tissues is restricted to mesothelial cells of the peritoneum, pleura, and pericardium, but MSLN is overexpressed in various solid tumors, including mesotheliomas, pancreatic cancers, triple-negative breast cancers (TNBC) and serous ovarian carcinomas [1, 2]. Additionally, MSLN has been shown to play a role in cell adhesion via its interaction with MUC16 (CA125), potentially facilitating peritoneal dissemination of cancer cells [3]. It has therefore emerged as a promising therapeutic target for the development of novel targeted therapies [4]. Among them, some have reached clinical trials, including immunotoxins such as SS1P [5–9] and LMB-100 [10, 11], antibody–drug conjugates like anetumab ravtansine [12–14], and cellular therapies [15, 16]. While some of these therapies, including SS1P and anetumab ravtansine, have shown encouraging responses, clinical trials have yet to establish consistent superiority over standard treatments, and none has yet reached approval [14]. Recent advances include the development of less immunogenic immunotoxins, as well as novel chimeric antigen receptor (CAR) T cells such as TC-210 and TC-510, which combine MSLN-targeted activity with immune checkpoint–modulating receptors [15, 16]. These investigational therapies aim to enhance efficacy, reduce toxicity, and improve clinical outcomes for patients with MSLN-expressing tumors.

As an alternative to these strategies, radio-theranostics could also be employed to target MSLN. This approach consists in selecting patients whose tumors express a specific target using nuclear imaging, followed by radioligand therapy using the same vector radiolabeled with a β^−^ or α emitter. The vector employed can be of different nature, including single-domain antibodies (sdAbs), also known as nanobodies^®^ or VHHs. SdAbs are antibody fragments derived from camelid heavy-chain-only antibodies and possess several advantageous properties for the development of radiopharmaceuticals, including high binding affinity, small size that facilitates tumor penetration, and rapid clearance that reduces systemic exposure [17, 18]. Some sdAbs have reached clinical trials in nuclear oncology as imaging agents, such as anti-HER2 [19], anti-PDL-1 [20], and anti-MMR [21] sdAbs. Among those, the anti-HER2 [^131^I]I-GMIB-Anti-HER2-VHH1 has also reached clinical trial radiolabeled with ^131^I for theranostic application [22].

A few anti-MSLN sdAbs are currently in preclinical development, such as [^99m^Tc]Tc-A1 for single photon emission computed tomography (SPECT) imaging [23, 24], and [^68^Ga]Ga-DOTA-A1 [25], [^68^Ga]Ga-NODAGA-S1 [26] and [^68^Ga]Ga-NOTA-269-H4 [27] for PET imaging. Recently, A1 derivatives have also been generated using directed mutagenesis. These sdAbs contain a single lysine residue, thereby allowing site-specific conjugation with the bispecific DOTA chelator. Among them, DOTA-A1K2 was selected as the lead candidate based on its radiolabeling stability and pharmacokinetic profile in mice following radiolabeling with ^68^Ga [28]. One advantage of using DOTA as the chelator is that it is well suited for theranostic applications since it allows radiolabeling with various radioisotopes, such as ^68^Ga for PET imaging and ^177^Lu or ^161^Tb for therapy.

^177^Lu, which has a 6.65-day half-life and combined β^−^ particles and gamma rays emissions, is commercially available in GMP grade and widely employed in routine clinical practice for radionuclide therapy of neuroendocrine tumors (NETs) and prostate cancers [29]. The use of [^177^Lu]Lu-DOTATATE (Lutathera) for peptide receptor radionuclide therapy (PRRT) in NETs is a prime example of the success of this theranostic approach with the NETTER-1 phase III clinical trial [30, 31]. The synergy between this radioisotope and ^68^Ga has established a robust platform for personalized medicine, allowing clinicians to tailor treatments based on imaging results, minimize systemic toxicity, and optimize therapeutic outcomes.

In contrast, ^161^Tb is an innovative radioisotope. It shares with ^177^Lu relatively similar half-life (6.96 days) and β^−^ particles energy, but additionally emits conversion and Auger electrons. Part of these low-energy electrons travel extremely short distances (< 500 nm), providing a concentrated cytotoxic effect against micro-metastases and residual tumor cells while minimizing collateral damages [32, 33]. Preclinical data indicated that ^161^Tb-based radioconjugates can improve tumor growth control compared to ^177^Lu analogs [34–36]. Clinical study of phase I/II are also performed like the first-in-human evaluation of [^161^Tb]Tb-PSMA-I&T in patients with metastatic castration-resistant prostate cancer [37].

The aim of this study was to radiolabel and evaluate the efficacy of [^177^Lu]Lu-DOTA-A1K2 and [^161^Tb]Tb-DOTA-A1K2 *in vitro* and *in vivo* in mice bearing human TNBC xenografts.

## Materials and methods

### Production and conjugation of DOTA-A1K2

SdAb A1K2 was produced and conjugated with DOTA as previously described [28]. Briefly, A1K2 was cloned into the pET-15b expression vector, then transformed into Shuffle T7 *E. coli*. Induction was performed with 1 mM isopropyl-β-d-thiogalactoside (IPTG) with incubation at 37 °C overnight. Purification involved immobilized metal affinity chromatography (IMAC) on Ni-NTA resin (Sigma-Aldrich) and gel filtration on a Superdex 75 h 16/60 column (Cytiva). The DOTA conjugation was performed using a fifty-fold molar excess of p-SCN-Bn-DOTA at pH 9. The mixtures were incubated for 3 h at 25 °C under stirring followed by filtration on a Superdex 75 h 10/30 column (Cytiva).

### DOTA-A1K2 radiolabeling with ^177^Lu and ^161^Tb

Radiolabeling with ¹⁷⁷Lu (^177^Lu^3+^ in aqueous 0.04 M HCl solution; ITM Radiopharma, Germany) was performed by mixing 100 µg of DOTA-A1K2-conjugated sdAb (7–8 nmol) with 150–200 µL of 0.1 M ammonium acetate buffer. Subsequently, 400–500 MBq of ¹⁷⁷LuCl₃ were added to adjust the pH to 5. The mixture was incubated at 60 °C for 30 min under continuous agitation. ¹⁶¹Tb was provided by the PRISMAP project, produced by irradiation of enriched ^160^Gd targets in the high flux reactors RHF (ILL, Grenoble, France) and BR2 (SCK CEN, Mol, Belgium) and radiochemically separated at SCK CEN. Radiolabeling with ¹⁶¹Tb (^161^Tb^3+^ in aqueous 0.05 M HCl solution) was performed using a similar pH, temperature, and incubation time. Following radiolabeling, unbound ¹⁷⁷Lu or ¹⁶¹Tb was removed by gel filtration on a NAP-5 column (Sephadex™ G-25 DNA Grade Gel, GE Healthcare) equilibrated with PBS (Sigma-Aldrich). The final solutions were sterile-filtered through a 0.22 μm Millex filter.

Radiochemical purity (RCP) was assessed both immediately after purification and after 24 h in the radiolabeling medium. Analyses were performed using a high-performance liquid chromatography (HPLC) system (Shimadzu) equipped with a radiodetector (Elysia-Raytest), using a C4 reverse-phase column (Symmetry300™, 5 μm, 150 × 4.6 mm, Waters). A gradient elution was carried out with solvent A (0.1% TFA in water) and solvent B (0.1% TFA in acetonitrile) under the following conditions: 0–1 min, 5% B; 1–5 min, linear increase from 5% to 90% B; 5–7 min, 90% B; 7–10 min, return to initial conditions; 10–12 min, 5% B at a flow rate of 1 mL/min.

### MSLN over-expression and lentiviral transduction

The MSLN sequence (TFORF0320, a gift from Feng Zhang; Addgene plasmid # 141582; http://n2t.net/addgene:141582; RRID: Addgene_141582 [38]) was subcloned into the pLenti6.3/TO/V5-Blasti vector (A11144, Thermo Fisher Scientific) to generate MSLN expression plasmids. Lentiviruses production was carried out by triple transfection of HEK-293T cells with the lentiviral transfer vector pLenti6.3/TO/V5-Blasti and the packaging plasmids psPAX2 (a gift from Didier Trono; Addgene plasmid #12260; http://n2t.net/addgene:12260; RRID: Addgene_12260) and pMD2.G (a gift from Didier Trono; Addgene plasmid #12259; http://n2t.net/addgene:12259; RRID: Addgene_12259), at a ratio of 0.3:0.3:0.1. Transfection was performed using jetPEI (101000053, Polyplus). Viral supernatants were collected 48 h after transfection, filtered through a 0.22 μm filter. MDA-MB-231 cells (ATCC: HTB-26) were transduced with lentiviral particles at a multiplicity of infection (MOI) of 0.3 in the presence of hexadimethrine bromide (4 µg/mL). LacZ was used for negative controls (MSLN-). Western Blot analysis was performed to confirm transfection using an anti-hMSLN antibody (Boster, PB9287) and β-actin (Cell Signaling, CST8457) as the loading control. Furthermore, MSLN expression was also controlled using FACS analysis on an Accuri C6 plus, using our sdAb A1K2-His as primary, revealed by anti-His-PE (Miltenyi Biotec). Data analysis was performed using FlowJo v10.10 software.

### Cell lines and culture conditions

Human breast cancer cell lines MDA-MB-231 (MSNL + and MSLN-), HCC70 (ATCC: CVCL_1270), HCC1806 (ATCC: CRL-2335) and the pancreatic adenocarcinoma cells line Capan-2 (ATCC: HTB-80) were employed. Cells were cultured in DMEM (PanBiotech, P04-03590) for MDA-MB-231, and RPMI 1640 medium (PanBiotech, P04-18047) for HCC70, HCC1806 and Capan-2. Both medium were supplemented with 10% fetal bovine serum (FBS; PanBiotech) and 1% penicillin–streptomycin (Dominic Dutscher). All cell lines were cultured less than 10 passages from thawed vials and maintained at 37 °C in 5% CO_2_ humidified incubator and mostly subcultured twice a week. A blasticidin antibiotic (Merck, SBR00022-1ML) selection was used at a concentration of 3 µg/ml for the MDA-MB-231 transfected cell lines.

### Animal models

Approval for all procedures was obtained from the animal care and ethics committee of Grenoble Alpes University and the French Ministry (APAFIS#19480–2019022616164184 v4). Mice had access to rodent chow and water *ad libitum*. They were maintained on a 12-hour light/dark cycle, with temperature between 20 and 24 °C and relative humidity of 40–60%. A one-week acclimatization period was observed prior to cells implantation.

Female athymic nude mice (Charles River, France; Janvier, France) at 5 weeks of age were subcutaneously xenografted with HCC70, HCC1806, Capan-2, or with MDA-MB-231 hMSLN + and MDA-MB-231 hMSLN- cells (3.5 × 10^6^ cells, in a 1:1 (v/v) PBS/Matrigel (Corning) mixture) in the hindlimbs. Tumor growth was monitored using a caliper 2 to 3 times a week, and tumor volume (V) was determine using the formula: $$\:V=L*l*h*\frac{\pi\:}{6}$$. For biodistribution studies, HCC70 or MDA-MB-231 tumors were allowed to grow for 3–4 weeks to reach approximately 100–300 mm³, and for efficacy studies, MDA-MB-231 tumors were allowed to grow for 2 weeks to reach approximately 40–80 mm^3^.

### Biodistribution studies

#### Gamma-well counting

Biodistribution studies were performed 2, 6, 24, 48, 72–144 h after intravenous injection *via* a tail vein with a low activity (1–3 MBq) of the [^177^Lu]Lu-DOTA-A1K2 or [^161^Tb]Tb-DOTA-A1K2. Gelofusin^®^ (100 µL, 4%) was co-administered 1–2 minutes before to reduce kidney retention. Stratified randomization ensured similar weights and tumor volumes between groups. Details of group sizes, mean mass, mean tumor volumes, and mean injected activities for each group can be found in the supplementary data (STables 1–3). Four mice were sacrificed for each time point by cervical dislocation, and tumors were harvested along with other organs. Tissues were weighed, and tracer activity was determined using a γ-counter (Wizard^2^, PerkinElmer). The results were corrected for decay, injected dose, and organ weight, and were expressed as a percentage of the injected dose per gram (%ID/g). Tumor-to-liver (T/L), tumor-to-kidney (T/K), and tumor-to-blood (T/B) activity ratios were computed. The time-activity curve for the kidneys, HCC70 tumors, liver and spleen were obtained with a bi-exponential function, fitted to the non-decay corrected data points between 2 and 168 h (Graphpad Prism 8.0).

#### SPECT/CT imaging

SPECT/CT acquisitions were performed on a dedicated subgroup of mice (*n* = 3) at 2, 6, 24, 48, 72 and 144 h after intravenous injection *via* a tail vein with higher activity (37 MBq) of the [^177^Lu]Lu-DOTA-A1K2 or [^161^Tb]Tb-DOTA-A1K2. SPECT/CT acquisitions were acquired under anesthesia with 2% isoflurane in 1:1 air: oxygen using dedicated system (nanoSPECT/CT Mediso). Images were corrected for decay and attenuation, and normalized to the injected dose.

### Efficacy studies

#### *In vitro* clonogenic cell survival assay

*In vitro* efficacy was evaluated using a clonogenic cell survival assay. Briefly, MDA-MB-231 (MSNL + and MSLN-) were seeded at 800 cells per well in 6-well plates. After 12 h, they were washed with PBS, and [^177^Lu]Lu-DOTA-A1K2 or [^161^Tb]Tb-DOTA-A1K2 were incubated for 24 h in culture medium at a concentration of 1 MBq/mL or 0.3 MBq/mL (*n* = 6 wells per condition). The cells were then washed thrice with PBS and cultured in medium for 6 days to allow colony formation. They were then washed with PBS and colored with Giemsa R (DDK Italia). The plates were photographed and the number of colonies determined using ImageJ software.

#### *In vivo* efficacy in mice bearing MSLN + tumors

Mice were randomized into five groups and treated with a single intravenous injection of [^177^Lu]Lu-DOTA-A1K2 or [^161^Tb]Tb-DOTA-A1K2. Two injected activities were evaluated for each, 10 MBq and 20 MBq. The control group received saline solution and all groups were co-injected with Gelofusin^®^ as described above. Tumor growth was monitored using a caliper 2 to 3 times per week, up to day 63. A second efficacy study was then conducted to evaluate the effect of lower doses of 2 and 5 MBq of either [^177^Lu]Lu-DOTA-A1K2 or [^161^Tb]Tb-DOTA-A1K2, using a similar protocol. Details of groups can be found in the supplementary data (STables 4–5). SPECT/CT imaging was performed in subgroups of mice of the 20 MBq groups at 24 h post injection, using the same method as described above.

#### Toxicity studies

During efficacy studies, total body mass was determined 2–3 times per week, and blood cell counts were determined 7 days before and 2 days after treatment using Thrombo-TIC, Ery-TIC and Leuco-TIC (Bioanalytic GmbH), before counting on Cellometer K2 (Nexcelom). At the end of the follow-up (63 days) kidneys, spleen, and liver were then frozen, 10 μm-thick slices were stained using hemato-eosin saffron (HES) and subsequently analyzed by a histopathologist. Serum creatinine and urea were measured (MScan-e, MSlabos).

### Statistical analysis

Mean values ± standard deviation (SD) were compared using nonparametric, unpaired tests: the Mann–Whitney U test for comparison of two groups and the Kruskal–Wallis test with Dunn’s correction for comparison of more than two groups. Tumor growth comparison analysis was made using two-way ANOVA with Geisser-Greenhouse correction, and followed with a Sidak’s multiple comparison test. Comparisons of biodistributions between [^177^Lu]Lu-DOTA-A1K2 and [^161^Tb]Tb-DOTA-A1K2 were made using ordinary two-way ANOVA, followed with a Sidak’s multiple comparison test of time and isotope effect. p values of 0.05 or lower were considered significant. Analyses were conducted with GraphPad Prism version 8.0.2.

## Results

### DOTA-A1K2 radiolabeling with ^177^Lu and ^161^Tb

**Fig. 1:**
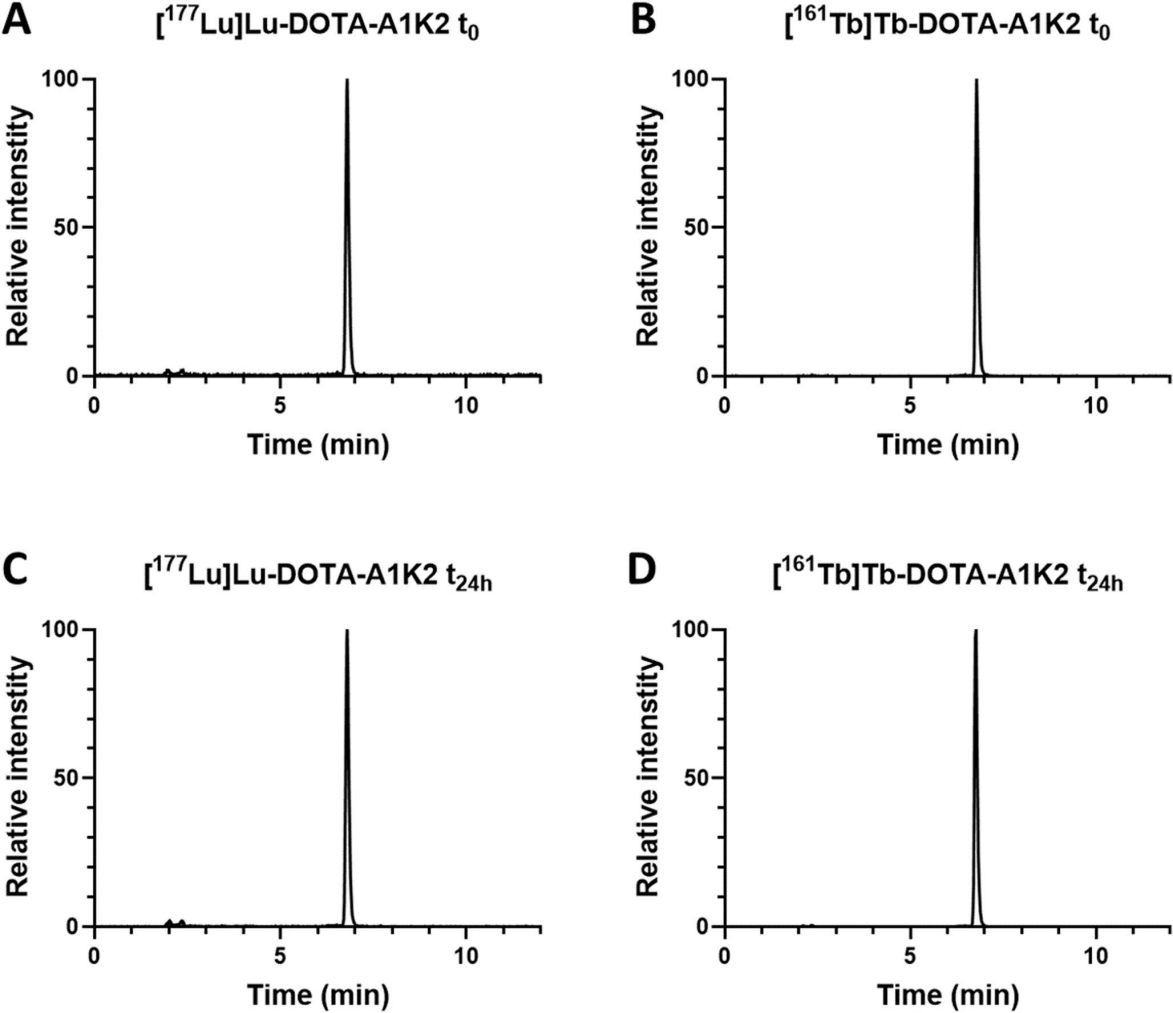
Radio-HPLC profiles of [^177^Lu]Lu-DOTA-A1K2 and [^161^Tb]Tb-DOTA-A1K2. Directly following radiolabeling and purification (**A** and **B**, respectively) and 24 h post-radiolabeling (**C** and **D**, respectively)

DOTA-A1K2 was successfully radiolabeled with ^177^Lu and ^161^Tb with RCPs of 98.0 ± 1.7% and 98.9 ± 1.2%, respectively (Fig. 1A and B). Moreover, both radiotracers remained stable for 24 h in the radiolabeling medium with RCPs of 97.4 ± 1.5% for [^177^Lu]Lu-DOTA-A1K2 and 98.3 ± 1.4% for [^161^Tb]Tb-DOTA-A1K2 (Fig. 1C and D).

### Comparison of [^177^Lu]Lu-DOTA-A1K2 and [^161^Tb]Tb-DOTA-A1K2 biodistribution *in vivo* in mice

**Fig. 2:**
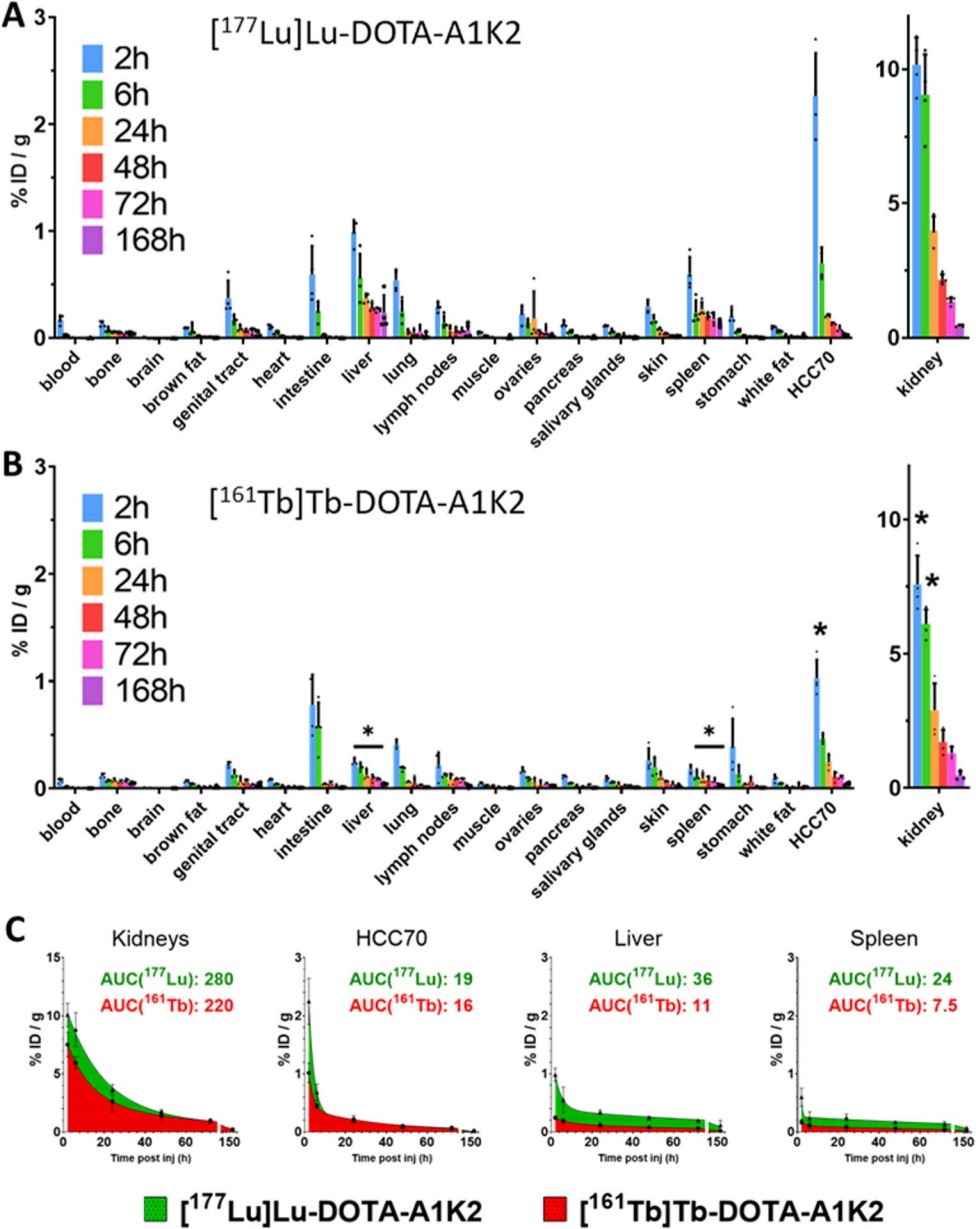
Comparison of [^177^Lu]Lu-DOTA-A1K2 and [^161^Tb]Tb-DOTA-A1K2 biodistribution *in vivo* in mice. **A**: Ex vivo biodistribution profile, 2 to 168 h after i.v. co-injection of [^177^Lu]Lu-DOTA-A1K2 (1–3 MBq) and Gelofusin, in mice bearing HCC70 xenografts (*n* = 4). **B**: Ex vivo biodistribution profile, 2 to 168 h after i.v. co-injection of [^161^Tb]Tb-DOTA-A1K2 (1–3 MBq) and Gelofusin, in mice bearing HCC70 xenografts (*n* = 4). *Significantly different from [^177^Lu]Lu-DOTA-A1K2 uptake (*p < 0.001). **C**: Area under the curve values of accumulated activity from 2 to 168 h p.i. (AUC2→168 h) calculated for [^161^Tb]Tb-DOTA-A1K2 (red) and [^177^Lu]Lu-DOTA-A1K2 (green)

To evaluate whether the choice of the radioisotope influences the biodistribution of DOTA-A1K2, its biodistribution was evaluated in mice bearing HCC70 tumors from 1 to 168 h following radiolabeling with either ^177^Lu or ^161^Tb (Fig. 2A-B). The area under the curve (AUC) was also determined in a subset of selected tissues using non-decay-corrected values to better reflect the accumulated activity (Fig. 2C). Ditto biodistribution profiles were observed in most investigated organs. In HCC70 tumors, [^161^Tb]Tb-DOTA-A1K2 uptake was significantly higher than [^177^Lu]Lu-DOTA-A1K2 at 2 h (*p* < 0.001), but no significant differences were found from 6 to 168 h. Consequently, tumor AUCs were comparable (19 vs. 16, respectively), with no significant differences. Thus, the isotope did not markedly impact tumor uptake, supporting side-by-side efficacy studies using matched injected activities.

However, significantly higher uptake of [^177^Lu]Lu-DOTA-A1K2 was observed in the liver and spleen (*p* < 0.001 vs. [^161^Tb]Tb-DOTA-A1K2) leading to > 3-fold increase in AUC. This difference may reflect subtle variations in the radiometal-chelate complex behavior, like stability, plasma protein binding, or minor differences in charge and coordination geometry between the ^177^Lu-DOTA and ^161^Tb-DOTA complexes [39]. Consequently, these two organs were included in the list of samples to be analyzed using histopathology during the efficacy studies to determine whether the choice of the radioisotope impacted toxicity in these off-target organs. In the kidney, the dose-limiting organ, a significant increase was also observed but to a lower extent (*p* < 0.001 at 2 and 6 h) so that the AUC was of 280 for [^177^Lu]Lu-DOTA-A1K2 and 220 for [^161^Tb]Tb-DOTA-A1K2. Uptakes in others organs are provided in Supplementary Tables (STable 6–9**)**.

These results also highlight that, despite the His-tag removal and the use of Gelofusin^®^, the tumor-to-kidney ratios remained suboptimal for therapy, suggesting that additional modifications remain necessary to further reduce kidney retention of DOTA-A1K2 in this animal model. The expression level of MSLN and hence DOTA-A1K2 uptake was found to be higher in other cell lines such as the Capan-2 and HCC1806 (SFig. 1). However, in order to investigate if ^161^Tb can improve DOTA-A1K2 efficacy in comparison to ^177^Lu, the selected strategy was to generate MDA-MD-231 cells transfected with MSLN. This allowed generation of an animal model with improved tumor targeting and permitted side-by-side *in vitro* and *in vivo* comparisons using MDA-MB-231 cells transfected with LacZ as negative controls.

### *In vitro* and *in vivo* evaluation of MDA-MB-231 MSLN + cells

**Fig. 3:**
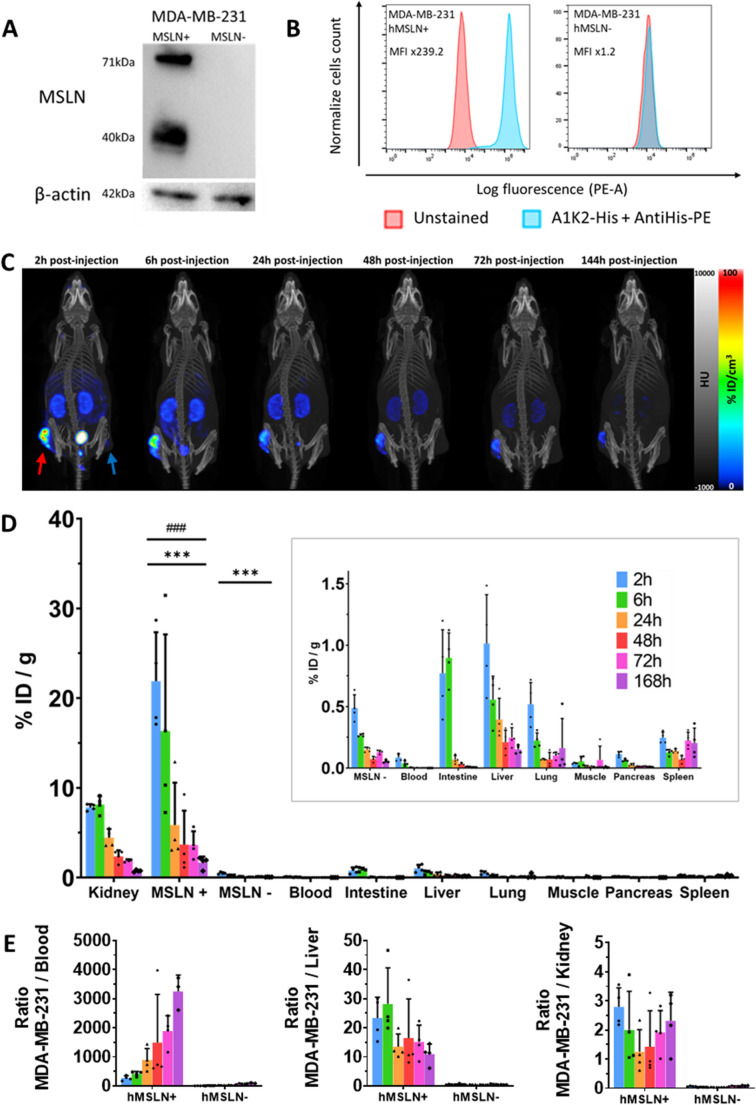
*In vitro* and *in vivo* evaluation of MDA-MB-231 MSLN + cells. **A**: MSLN expression of transfected MDA-MB-231 cells was confirmed by Western Blot. **B**: FACS analysis confirmed MSLN expression of transfected MDA-MB-231 cells. MFI = Median Fluorescence Intensity. **C**: Representative SPECT/CT images of one representative mouse bearing MDA-MB-231 MSLN+ (red arrow) and MSLN- (blue arrow) xenografts, 2 to 144 h after i.v. co-injection of [^161^Tb]Tb-DOTA-A1K2 and Gelofusin (*n* = 3). **D**: Ex vivo biodistribution profile of mice bearing both MDA-MB-231 MSLN + and MSLN- xenografts, 2 to 168 h after i.v. injection (*n* = 4) ***p < 0.001 vs. kidneys. ^###^*p* < 0.001 vs. MSLN- cells (*n* = 4 per time point). Insert: focus on organs ranging between 0 and 1.5%ID/g. **E**: Tumor-to-blood, tumor-to-liver, and tumor-to-kidney ratios determined from 2 to 168 h after i.v. injection (*n* = 4 per time point)

MSLN expression in transfected MDA-MB-231 cells was confirmed by western blot, where MSLN precursor (71 kDa) and MSLN (40 kDa) expression were observed, while no signal was detectable in LacZ-transfected controls (Fig. 3A). FACS analysis confirmed the efficiency of the transfection, with a 239-fold increase in mean fluorescent intensity in MSLN + cells, while no difference was observed in MSLN- cells (Fig. 3B). *In vivo* in nude mice grafted with MDA-MB-231 MSLN + cells, the tumor was readily visible up to 144 h post-injection by SPECT using [^161^Tb]Tb-DOTA-A1K2. Moreover, the intensity of the signal observed in the tumor was greater than that observed in the kidneys at all time points (Fig. 3C). Biodistribution quantifications, performed by gamma-well counting (Fig. 3D), confirmed that the highest uptake was observed in the MSLN + tumors at all investigated time points, ranging from 21.9 ± 5.5%ID/g at 2 h to 1.7 ± 0.7%ID/g at 168 h. This uptake was significantly higher than that of the kidneys, which ranged from 7.8 ± 0.4%ID/g at 2 h to 0.7 ± 0.1%ID/g at 168 h (*p* < 0.001 vs. MSLN+). MSLN + tumor-to-kidney ratios were therefore always > 1, with a maximum of 2.8 ± 0.6 at 2 h (Fig. 3E). With the exception of the tumor and kidneys, the uptake was found to be ≤ 1%ID/g in all other investigated organs and at all time points, including the MSLN- tumors where it ranged from 0.5 ± 0.1%ID/g at 2 h to 0.1 ± 0.0%ID/g at 168 h. Uptake in MSLN + tumors was therefore significantly higher than that of MSLN- tumors, thereby demonstrating the specificity of the signal (*p* < 0.001 vs. MSLN+). Elevated tumor-to-blood, tumor-to-liver and tumor-to-kidney ratios were therefore observed (Fig. 3E). Uptake in other organs is provided in Supplementary Tables (STable 10–11).

### *In vitro* and *in vivo* comparison of [^177^Lu]Lu-DOTA-A1K2 and [^161^Tb]Tb-DOTA-A1K2 efficacies

**Fig. 4:**
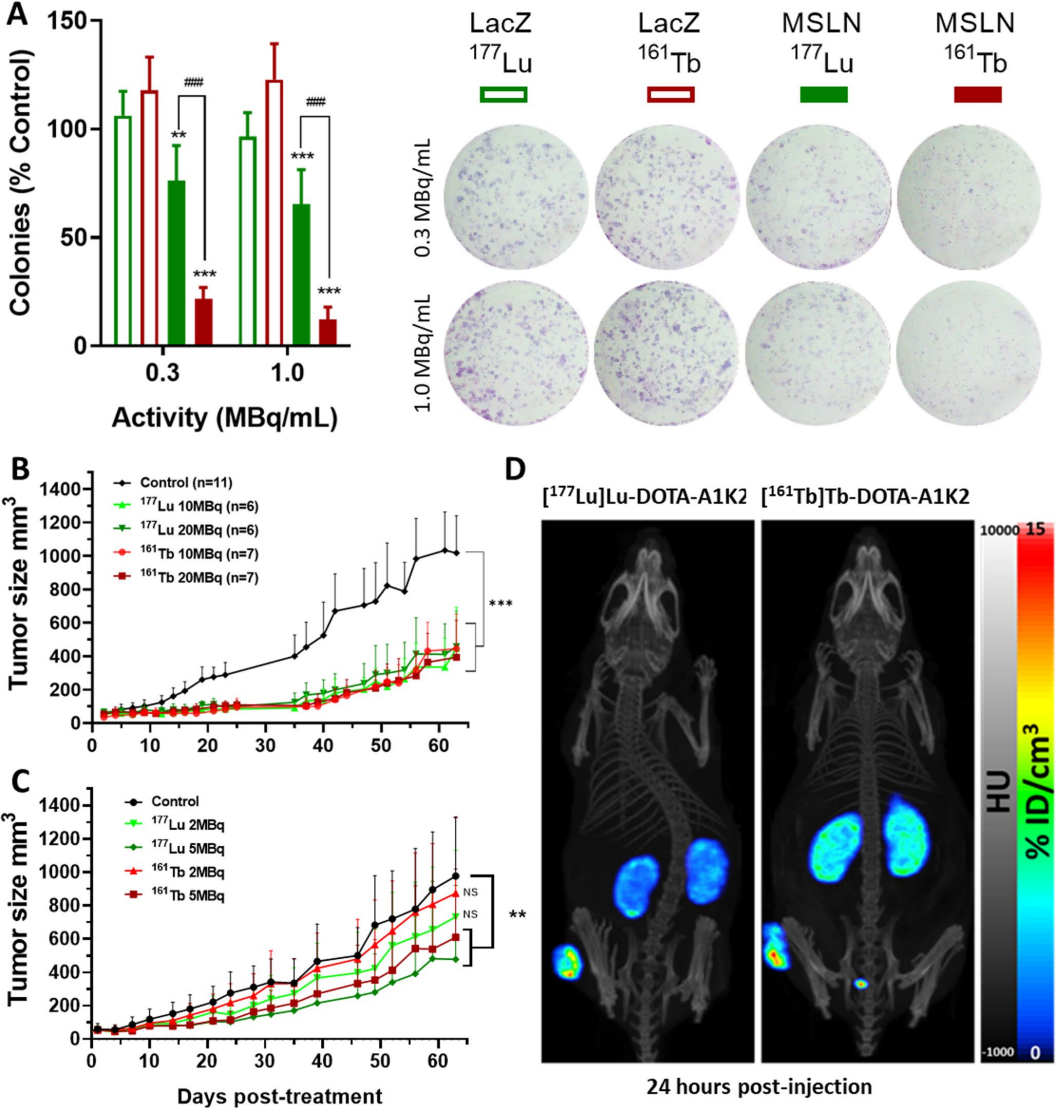
*In vitro* and *in vivo* comparison of [^177^Lu]Lu-DOTA-A1K2 and [^161^Tb]Tb-A1K2 efficacies. **A**: Clonogenic assay showing the fold of reduction in the number of colonies (as a percentage of the untreated control) after incubation with two different activities of [^161^Tb]Tb-DOTA-A1K2 or [^177^Lu]Lu-DOTA-A1K2. Representative images of colonies colored with Giemsa. **p < 0.01 and ***p < 0.001 vs. MSLN-. ^###^*p* < 0.001 vs ^161^Tb. **B**: MDA-MB-231 MSLN + tumor growth in mice treated with 10 or 20 MBq of [^177^Lu]Lu-DOTA-A1K2 (light and dark green, *n* = 6), 10 or 20 MBq of [^161^Tb]Tb-DOTA-A1K2 (light and dark red, *n* = 7), or saline solution (black, *n* = 11). ***p < 0.001 vs. control group. **C**: MDA-MB-231 MSLN + tumor growth in mice treated with 2 or 5 MBq of [^177^Lu]Lu-DOTA-A1K2 (light and dark green), 2 or 5 MBq of [^161^Tb]Tb-DOTA-A1K2 (light and dark red), or saline solution (black) (*n* = 12 for all). **p < 0.01 vs. control group. **D**: Representative SPECT/CT images of mice 24 h after a single injection of 20 MBq of [^177^Lu]Lu-DOTA-A1K2 (left) or [^161^Tb]Tb-DOTA-A1K2 (right)

The efficacy of [^177^Lu]Lu-DOTA-A1K2 and [^161^Tb]Tb-DOTA-A1K2 on MSLN + cells was first evaluated *in vitro* using clonogenic cell survival assay (Fig. 4A). No effect was observed on MSLN- cells, while significant reduction in the number of colonies was observed for both radiotracers on MSLN + cells at doses of 1 and 0.3 MBq/mL. This effect was significantly higher for [^161^Tb]Tb-DOTA-A1K2, with 88% and 78% reduction in colony number at 1 and 0.3 MBq/mL, respectively (vs. 35% and 24% reductions for [^177^Lu]Lu-DOTA-A1K2, *p* < 0.001).

The efficacy was then evaluated *in vivo* in mice bearing MSLN + tumors. First, single intravenous injection of 20 or 10 MBq doses of either [^177^Lu]Lu-DOTA-A1K2 or [^161^Tb]Tb–A1K2 were evaluated in comparison to control group treated with saline. Both radiotracers significantly inhibited tumor growth up to 35 days p.i. (*p* < 0.001 vs. control). However, no differences were observed between the two evaluated doses or between the two radioisotopes (p = not significant) (Fig. 4B). Individual tumor growths are available in supplementary data (SFig. 2).

The lack of differences between the two evaluated doses might be attributable to the fact that the maximal response was achieved using the lowest dose of 10 MBq, which could also have impaired the comparison between the two radioisotopes. Therefore, a second efficacy study was conducted using lower doses of 2 or 5 MBq (Fig. 4C). A significant inhibition of tumor growth was observed in the two groups injected with the highest dose of 5 MBq compared to control group (*p* < 0.001), while the lowest dose of 2 MBq failed to significantly inhibit growths in both treated groups. Similarly, as previously observed using 10 MBq and 20 MBq doses, no differences were observed between the groups injected with [^177^Lu]Lu-DOTA-A1K2 or [^161^Tb]Tb-DOTA-A1K2. Individual tumor growths are available in supplementary data (SFig. 3). Kaplan-Meier survival curves confirmed those significant observations (SFig. 4). SPECT/CT acquisitions performed at 24 h in a subgroup of mice treated with the 20 MBq dose confirmed that the tumor showed the highest uptake at this time point, comparable between [^177^Lu]Lu-DOTA-A1K2 and [^161^Tb]Tb-DOTA-A1K2 (Fig. 4D).

**Fig. 5:**
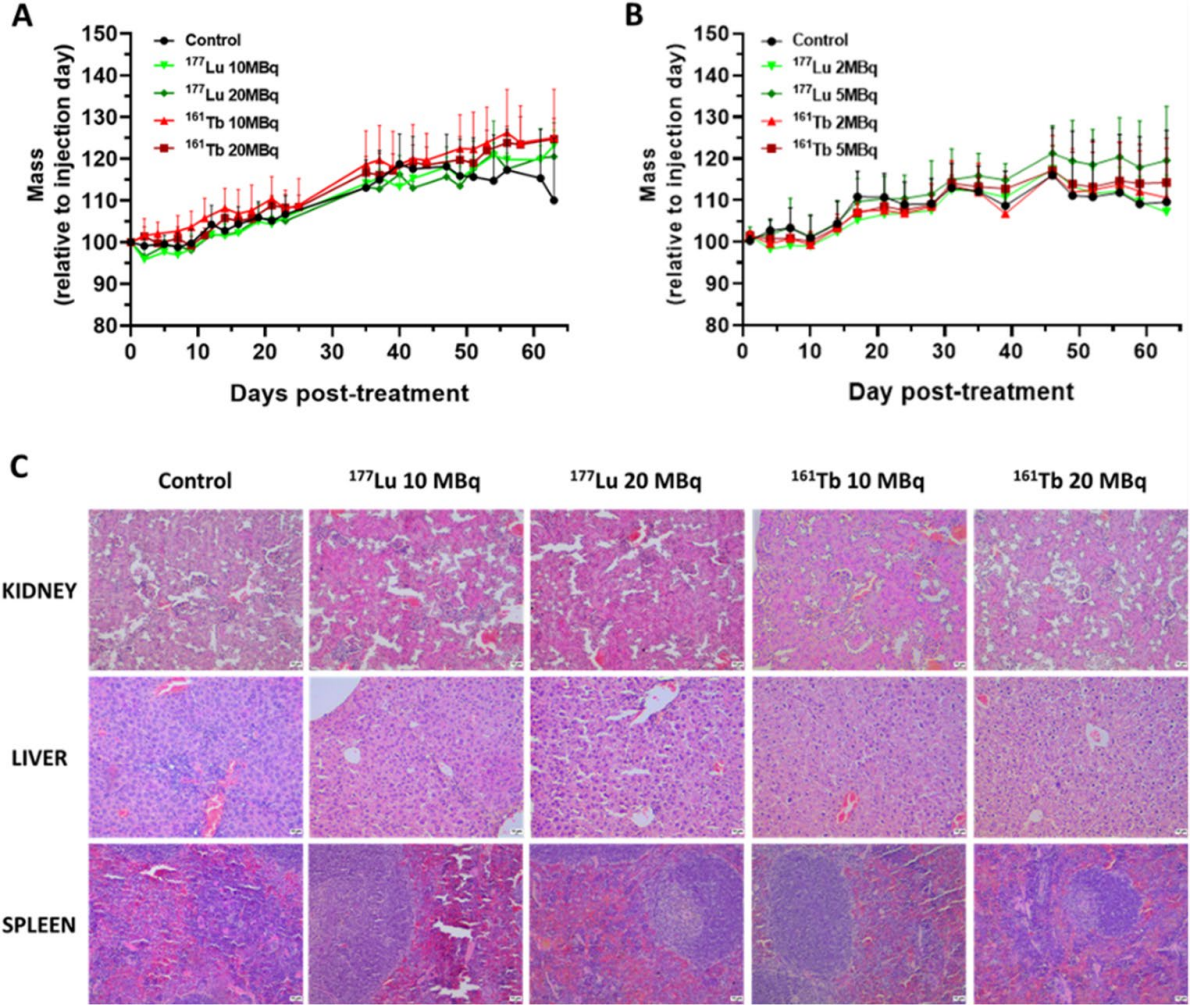
*In vivo* toxicity study from the mice treated with [^177^Lu]Lu-DOTA-A1K2 or [^161^Tb]Tb-DOTA-A1K2. **A**: Total body mass of mice relative to the injection day. Mice were treated with 10 or 20 MBq of [^177^Lu]Lu-DOTA-A1K2 (light and dark green, *n* = 6), or with 10 or 20 MBq of [^161^Tb]Tb-DOTA-A1K2 (light and dark red, *n* = 7), or saline solution (black, *n* = 11). No statistical differences were observed. **B**: Total body mass of mice relative to the injection day. Mice were treated with 2 or 5 MBq of [^177^Lu]Lu-DOTA-A1K2 (light and dark green, *n* = 12), or with 2 or 5 MBq of [^161^Tb]Tb-DOTA-A1K2 (light and dark red, *n* = 12), or saline solution (black, *n* = 12). No statistical differences were observed. **C**: Kidneys, liver and spleen histology. At day 63, the end of the follow-up period, sections of organs were stained with standard HES trichrome. The architecture of the organ was found to be preserved, with no sign of fibrosis or inflammation or degradation

Regarding the safety, no change was observed during the follow-up in the total body mass (Fig. 5A and B) and no off-target effect, such as inflammation, fibrosis, or architecture alteration, was observed on HES-stained kidneys, liver and spleen sections (Fig. 5C) across all groups at the final time point (63 days). Renal biomarkers, including plasma urea and creatinine concentrations, were within the normal physiological range, as were the hematological parameters assessed in the complete blood counts (SFigs. 5 and 6).

## Discussion

The aim of the present study was to compare the efficacy of the sdAbs [^177^Lu]Lu-DOTA-A1K2 and [^161^Tb]Tb-DOTA-A1K2 directed against MSLN. DOTA-A1K2 was successfully radiolabeled with either ^177^Lu or ^161^Tb with RCPs > 95%. *In vivo* in mice bearing HCC70 tumors, they exhibited comparable activity accumulation within the tumor. However, suboptimal tumor-to-kidney ratios precluded the use of the same animal model to compare their efficacy. Therefore, MSLN negative MDA-MB-231 cells were transfected with MSLN to generate a model with improved tumor uptake, allowing investigation of the hypothesis that ^161^Tb, which emits conversion and Auger electrons in addition to β^−^ particles, could provide additional effects compared to ^177^Lu. MSLN transfection led to improve *in vivo* pharmacokinetics with maximal uptake of [^161^Tb]Tb-A1K2 observed in MDA-MB-231 MSLN + tumors at all investigated time points. *In vitro*, both [^177^Lu]Lu-DOTA-A1K2 and [^161^Tb]Tb-DOTA-A1K2 were found to significantly reduce MSLN + cell survival in a clonogenic cell survival assay. Moreover, [^161^Tb]Tb-DOTA-A1K2 was significantly more potent than [^177^Lu]Lu-DOTA-A1K2. However, *in vivo* in mice bearing MSLN + cells, while both [^177^Lu]Lu-DOTA-A1K2 and [^161^Tb]Tb-DOTA-A1K2 accumulated in tumors and significantly reduced tumor growth following a single injection of 5, 10 or 20 MBq, no such superiority of ^161^Tb over ^177^Lu was observed, suggesting that in this model the additional Auger electron contribution from ^161^Tb does not translate into a measurable therapeutic gain.

Auger electrons are typically considered to be cytotoxic when emitted near nuclear DNA, therefore internalization and nuclear proximity are critical to maximize their therapeutic effect [40]. Indeed, previous studies targeting strongly internalizing antigens like PSMA have demonstrated marked superiority of ^161^Tb-labeled compared to ^177^Lu-labeled agents [36]. However, the need for ^161^Tb to be internalized to demonstrate a superiority over ^177^Lu remains controversial. Indeed, recent findings have demonstrated that non-internalized peptides, bound to the membrane and radiolabeled with ^161^Tb, such as [^161^Tb]Tb-DOTA-LM3, a non-internalized somatostatin antagonist, significantly outperformed its ^177^Lu radiolabeled counterpart, [^177^Lu]Lu-DOTA-LM3, in terms of therapeutic efficacy, despite its extracellular localization [41]. This unexpected observation implies a potential “membrane effect”, where cell membrane targeting may activate alternative cytotoxic mechanisms, challenging the previously established requirement of nuclear localization for exploiting the therapeutic potential of short-ranged electrons [42, 43]. Recent clinical studies with [^161^Tb]Tb-DOTATOC and [^161^Tb]Tb-PSMA-I&T have demonstrated the feasibility and safety of ^161^Tb-based therapies [37, 44]. Our work extends this perspective to sdAb-based vectors targeting mesothelin.

Regarding the sdAb A1, from which DOTA-A1K2 is derived, previous studies have reported its internalization *in vitro* using fluorescent probes [26, 45]. However, the extent of internalization *in vivo* remains unclear. In a previous study, we have demonstrated that at least 50% of [^68^Ga]Ga-DOTA-A1K2 uptake in HCC70 tumors at 1 h remains un-internalized, as evidenced by an *in vivo* displacement assay [28].

While the *in vitro* efficacy essay, that was conducted in a “single-cell” situation, might have favored the efficacy of the Auger electron over the beta-minus emission in comparison to the *in vivo* situation, the absence of *in vivo* difference in between [^161^Tb]Tb-DOTA-A1K2 and [^177^Lu]Lu-DOTA-A1K2 was unexpected. During the past few years, MSLN shedding has been reported. MSLN undergoes proteolytic cleavage predominantly via ADAM family metalloproteases, releasing soluble fragments into the tumor matrix and ultimately to the circulation, leaving only a short membrane-anchored stalk on tumor cells [46]. Since the released fragment includes the epitope of the sdAb A1, we hypothesized that this mechanism can be responsible not only for a reduction of A1 internalization *in vivo*, but also for its binding to sites located a short distance from the tumor cell membrane. Such binding to the shed forms of MSLN could even lead to lower tumor retention. This mechanism is even more problematic for therapies requiring a direct binding to tumor cell membranes, such as ADC and CAR-T cells. As a matter of fact, it has been reported that shedding reduces the efficacy of various mesothelin-targeted therapies (immunotoxins, CAR-T cells), and eventually the inhibition of shedding with protease inhibitors like marimastat significantly enhances their effectiveness [47, 48].

To overcome this limitation, future anti-MSLN sdAbs production could focus on selecting novel sdAbs with epitopes located within the stable juxtamembrane segment of mesothelin. Targeting this region avoids interaction with soluble mesothelin, potentially prolonging tumor cell membrane retention and increasing internalization. While large targeting molecules (like conventional antibodies or CAR receptors) can suffer steric hindrance at these membrane-proximal epitopes, smaller sdAbs have fewer steric constraints, maintaining high affinity and tumor specificity. Recent studies, including CAR-T cells targeting this juxtamembrane segment (like 15B6 scFv), confirmed that this strategy reduces shedding-related efficacy losses and enhances tumor retention, however reporting affinity challenges due to steric constraints [48]. SdAbs could therefore offer an elegant solution, potentially delivering radionuclides more effectively to the cell surface or intracellular compartments following endocytic uptake.

This study highlights the necessity of engineering sdAbs that not only exhibit high binding affinity but also optimal localization and retention for theranostics application, as well as the need to tailor the choice of the radioisotope accordingly. In addition to generating novel sdAbs directed at juxtamembrane epitopes that might better exploit from ^161^Tb characteristics, further optimization of the A1K2 sdAb can also be performed to improve its therapeutic potential when labeled with ^177^Lu. In comparison to the original version of A1 labeled with ^99m^Tc, renal biodistribution properties for DOTA-A1K2 has already been significantly improved following removal of myc and poly-his tags, and using co-administration of Gelofusin^®^ [49]. Despite this, kidneys remain the dose limited organ and can preclude therapeutic approach [50]. Future studies will therefore focus on additional mitigation strategies, including the introduction of kidney-specific cleavable linkers [51], pretargeting approaches [52] or co-injection of amino acid solutions [53], to further lower renal dose.

The use of a MSLN + and MSLN- transfected cell lines was well suited for the *in vitro* and *in vivo* comparison of ^177^Lu- versus ^161^Tb-labeled DOTA-A1K2 efficacy using optimized and controlled models with elevated MSLN expression. However, further studies will be necessary to refine the therapeutic protocol using more clinically relevant models. This will include the use of cell lines endogenously expressing MSLN, such as HCC1806 or PDx, as well as the evaluation of fractionated dose administration. These additional studies will also allow further evaluating chronic toxicity. Indeed, while the present study showed an absence of renal toxicity at 9 weeks, as determined by blood analysis and histology, the kidney therapeutic index of sdAb remains poor [52] and long-term toxicity has sometimes been reported at later time points [54].

## Conclusion

This study provides an *in vitro* and *in vivo* comparative assessment of [^177^Lu]Lu-DOTA-A1K2 and [^161^Tb]Tb-DOTA-A1K2 therapeutic efficacy. *In vivo* in mice bearing MSLN-positive tumors, both radiotracers successfully inhibited tumor growth. The *in vitro* superiority of ^161^Tb, that could be attributable to its emission of conversion and Auger electrons, did not translate into a clear advantage *in vivo*. This may be due to DOTA-A1K2 binding to the shed forms of mesothelin, and further studies with protease inhibitors will be mandatory to test this hypothesis, by evaluating their impact on MSLN expression by tumor cells and on soluble MSLN expression within the tumor micro-environment. A better understanding of possible MSLN shedding and redistribution will also be important for accurate micro-dosimetric calculations. Future improvements including selection of a next-generation anti-MSLN sdAbs directed against non-shed, membrane-proximal epitopes, will also allow testing this hypothesis *in vivo* and could result in enhanced therapeutic efficacy.

## Supplementary Information

Below is the link to the electronic supplementary material.

Supplementary Material 1

## Data Availability

The datasets generated and analysed during the current study are available from the corresponding author on reasonable request.

## Abbreviations

%ID/g: percentage of injected dose per gram
^161^Tb: Terbium-161
^177^Lu: Lutetium-177
^68^Ga: Gallium-68
ADC: antibody drug conjugate
CAR-T: Chimeric antigen receptor T cell
CD: circular dichroism
CT: computed tomography
DOTA: 1,4,7,10-tetraazacyclododecane-1,4,7,10-tetraacetic acid
FACS: fluorescence activated cell sorting
GPI: glycosylphosphatidylinositol
HES: hematoxylin-eosin-saffron
HER2: human epidermal growth factor receptor 2
HPLC: high-pressure liquid chromatography
IMAC: immobilized metal affinity chromatography
MMR: macrophage mannose receptor
MSLN: mesothelin
NETs: neuroendocrine tumors
NOTA: 2,2′,2”-(1,4,7-triazacyclononane-1,4,7-triyl)triacetic acid
PBS: phosphate buffer saline
PDCA: pancreatic ductal adenocarcinoma
PDL-1: programmed death-ligand 1
PET: positron emission tomography
PSMA: prostate specific membrane antigene
RCP: radiochemical purities
sdAb: single domain antibody
SPECT: single photon emission computed tomography
TFA: trifluoroacetic acid
TNBC: triple negative breast cancer
VHH: variable regions of the heavy chain of heavy-chain-only antibodies
