# Supplementary material for: Comparative theranostic efficacy of 177Lu- and 161Tb-labeled A1K2 SdAb in mesothelin-positive tumors
Source: Eur J Nucl Med Mol Imaging. 2025 Dec 27;53(6):3775–88. doi: 10.1007/s00259-025-07723-z (PMC13121286; doi:10.1007/s00259-025-07723-z)
Supplement: Supplementary file 1 — Supplementary Material 1 [file 259_2025_7723_MOESM1_ESM.docx]

**Comparative theranostic efficacy of ^177^Lu- and ^161^Tb-labeled A1K2 sdAb in mesothelin-positive tumors**

**Author names and affiliations**

*Émilien N’Guessan^1^, Florian Raes^1^, Mitra Ahmadi^1^, Sandrine Bacot^1^, Laurent Dumas^1^, Julien Leenhardt^1,2^, Amaury du Moulinet d’Hardemare^3^, Marlène Debiossat^1^, Clémence André^1^, Jean Boutonnat^4^, Jérôme Durivault^5^, Christopher Montemagno^5^, Jean-Luc Lenormand^6^, Loïc Djaïleb^1^, Ulli Köster^7^, Michiel Van de Voorde^8^, Stijn Ramaekers^8^, Ken Verguts^8^, Catherine Ghezzi^1^, Pascale Perret^1^, Charlotte Lombardi^1, 6#^, Alexis Broisat^1#^*

*^1^ Univ. Grenoble Alpes, INSERM U1039, LRB, 38000 Grenoble, France*

*^2^ Univ. Grenoble Alpes, INSERM U1039, CHU Grenoble Alpes, LRB, Département de Médecine Nucléaire, 38000 Grenoble, France*

^3^ *Univ. Grenoble Alpes, CNRS 5250, DCM, 38000 Grenoble, France*

^4^ *Univ. Grenoble Alpes, CHU Grenoble Alpes, Département de Pathologie, 38000 Grenoble, France*

*^5^ Biomedical Department, Centre Scientifique de Monaco, 98000 Monaco, Principality of Monaco*

*^6^ Univ. Grenoble Alpes, CNRS 5525, TIMC-Tree, 38000 Grenoble, France*

*^7^ Institut Laue-Langevin, 38000 Grenoble, France*

*^8^ SCK CEN, Nuclear Medical Applications, Mol, Belgium*

# Contributed equally to this work

Corresponding Author: Alexis Broisat, [alexis.broisat@inserm.fr](mailto:)

**Supplementary**

**Table 1.** Details of the different groups of mice bearing HCC70 tumors injected with the **[^177^Lu]Lu-DOTA-A1K2** sdAb for the *in vivo* biodistribution study over time. Expressed as mean ± SD (standard deviation) (n=4)

|  | **2h p.i.** | | | **6h p.i.** | | | **24h p.i.** | | | **48h p.i.** | | | **72h p.i.** | | | **168h p.i.** | | | |
| --- | --- | --- | --- | --- | --- | --- | --- | --- | --- | --- | --- | --- | --- | --- | --- | --- | --- | --- | --- |
|  | Mean | ± | SD | Mean | ± | SD | Mean | ± | SD | Mean | ± | SD | Mean | ± | SD | Mean | ± | SD |  |
| ID t inj (MBq) | 1.26 | ± | 0.06 | 1.27 | ± | 0.06 | 1.52 | ± | 0.05 | 1.52 | ± | 0.05 | 1.43 | ± | 0.19 | 1.44 | ± | 0.16 |  |
| Mass (g) | 19.36 | ± | 0.67 | 20.82 | ± | 0.90 | 19.81 | ± | 0.59 | 20.70 | ± | 0.66 | 20.05 | ± | 0.31 | 21.48 | ± | 1.45 |  |
| Tumor HCC70  size (mm^3^) | 274.45 | ± | 48.24 | 233.28 | ± | 25.72 | 261.39 | ± | 73.07 | 289.86 | ± | 101.09 | 253.31 | ± | 85.33 | 213.28 | ± | 86.02 |  |

|  | **2h p.i.** | | | **6h p.i.** | | | **24h p.i.** | | | **48h p.i.** | | | **72h p.i.** | | | **168h p.i.** | | |
| --- | --- | --- | --- | --- | --- | --- | --- | --- | --- | --- | --- | --- | --- | --- | --- | --- | --- | --- |
|  | Mean | ± | SD | Mean | ± | SD | Mean | ± | SD | Mean | ± | SD | Mean | ± | SD | Mean | ± | SD |
| ID t inj (MBq) | 2.12 | ± | 0.04 | 2.19 | ± | 0.12 | 2.15 | ± | 0.05 | 2.10 | ± | 0.27 | 2.10 | ± | 0.07 | 2.15 | ± | 0.09 |
| Mass (g) | 19.68 | ± | 1.60 | 18.53 | ± | 1.04 | 15.64 | ± | 9.28 | 19.86 | ± | 0.98 | 18.78 | ± | 2.68 | 15.41 | ± | 9.60 |
| Tumor HCC70 size (mm^3^) | 133.16 | ± | 80.09 | 136.91 | ± | 56.90 | 115.97 | ± | 36.56 | 124.71 | ± | 37.15 | 121.95 | ± | 33.17 | 113.44 | ± | 24.27 |

**Table 2.** Details of the different groups of mice bearing HCC70 tumors injected with the [**^161^Tb]Tb-DOTA-A1K2** sdAb for the *in vivo* biodistribution study over time. Expressed as mean ± SD (standard deviation) (n=4)

**Table 3.** Details of the different groups of mice bearing MDA-MB-231 hMSLN+ or - tumors injected with the [**^161^Tb]Tb-DOTA-A1K2** sdAb for the *in vivo* biodistribution study over time. Expressed as mean ± SD (standard deviation) (n=4)

|  | **2h p.i.** | | | **6h p.i.** | | | | **24h p.i.** | | | | **48h p.i.** | | | | **72h p.i.** | | | | **168h p.i.** | | |
| --- | --- | --- | --- | --- | --- | --- | --- | --- | --- | --- | --- | --- | --- | --- | --- | --- | --- | --- | --- | --- | --- | --- |
|  | Mean | ± | SD | | Mean | ± | SD | | Mean | ± | SD | | Mean | ± | SD | | Mean | ± | SD | Mean | ± | SD |
| ID t inj (MBq) | 3.04 | ± | 0.07 | | 3.00 | ± | 0.25 | | 2.92 | ± | 0.21 | | 3.00 | ± | 0.52 | | 2.94 | ± | 0.04 | 2.73 | ± | 0.35 |
| Mass (g) | 19.33 | ± | 1.32 | | 19.43 | ± | 0.64 | | 18.03 | ± | 0.51 | | 20.03 | ± | 0.80 | | 19.35 | ± | 1.11 | 18.83 | ± | 0.83 |
| Tumor MDA-MB-231 hMSLN+  size (mm^3^) | 56.73 | ± | 14.77 | | 68.08 | ± | 18.94 | | 68.51 | ± | 9.64 | | 99.02 | ± | 11.52 | | 57.53 | ± | 9.68 | 60.35 | ± | 18.52 |
| Tumor MDA-MB-231 hMSLN-  size (mm^3^) | 114.18 | ± | 43.27 | | 97.40 | ± | 34.33 | | 83.82 | ± | 47.38 | | 87.70 | ± | 36.83 | | 85.91 | ± | 28.15 | 112.03 | ± | 43.40 |

**Table 4. Design of the tumor therapy study with high activity**

| Radiotracers | **Activity (MBq)** | **Number of mice (n)** | **Initial tumor volume (mm^3^)** | **Initial body weight (g)** |
| --- | --- | --- | --- | --- |
| Vehicle (control) | / | 11 | 50.5 ± 27.2 | 19.6 ± 1.1 |
| [^161^Tb]Tb-DOTA-A1K2 | 10 | 7 | 42.1 ± 19.2 | 18.8 ± 1.2 |
| [^161^Tb]Tb-DOTA-A1K2 | 20 | 7 | 60.9 ± 25.3 | 19.4 ± 0.7 |
| [^177^Lu]Lu-DOTA-A1K2 | 10 | 6 | 52.0 ± 17.3 | 20.5 ± 1.1 |
| [^177^Lu]Lu-DOTA-A1K2 | 20 | 6 | 53.5 ± 7.6 | 20.6 ± 0.6 |

**Table 5. Design of the tumor therapy study with low activity**

| Radiotracers | **Activity (MBq)** | **Number of mice (n)** | **Initial tumor volume (mm^3^)** | **Initial body weight (g)** |
| --- | --- | --- | --- | --- |
| Vehicle (control) | / | 12 | 52.2 ± 29.3 | 19.1 ± 1.3 |
| [^161^Tb]Tb-DOTA-A1K2 | 2 | 12 | 48.2 ± 23.2 | 19.2 ± 0.8 |
| [^161^Tb]Tb-DOTA-A1K2 | 5 | 12 | 51.4 ± 20.0 | 18.8 ± 0.7 |
| [^177^Lu]Lu-DOTA-A1K2 | 2 | 12 | 48.3 ± 20.0 | 19.2 ± 1.1 |
| [^177^Lu]Lu-DOTA-A1K2 | 5 | 12 | 48.7 ± 15.0 | 18.1 ± 1.0 |

**Table 6.** *Ex vivo* biodistribution data obtained in mice bearing HCC70 tumors injected with the **[^177^Lu]Lu-DOTA-A1K2** sdAb, at different time points post-injection (p.i.). Expressed as mean ± SD (standard deviation) (n=4)

|  | **2h p.i.** | | | **6h p.i.** | | | **24h p.i.** | | | **48h p.i.** | | | **72h p.i.** | | | **168h p.i.** | | | |
| --- | --- | --- | --- | --- | --- | --- | --- | --- | --- | --- | --- | --- | --- | --- | --- | --- | --- | --- | --- |
| Organs | Mean | ± | SD | Mean | ± | SD | Mean | ± | SD | Mean | ± | SD | Mean | ± | SD | Mean | ± | SD |  |
| Adrenals | 0.23 | ± | 0.03 | 0.13 | ± | 0.02 | 0.08 | ± | 0.02 | 0.05 | ± | 0.01 | 0.09 | ± | 0.02 | 0.06 | ± | 0.08 |  |
| Blood | 0.17 | ± | 0.04 | 0.03 | ± | 0.01 | 0.01 | ± | 0.00 | 0.00 | ± | 0.00 | 0.00 | ± | 0.00 | 0.00 | ± | 0.00 |  |
| Bone | 0.14 | ± | 0.03 | 0.08 | ± | 0.02 | 0.06 | ± | 0.01 | 0.05 | ± | 0.01 | 0.05 | ± | 0.01 | 0.05 | ± | 0.01 |  |
| Brain | 0.01 | ± | 0.00 | 0.00 | ± | 0.00 | 0.00 | ± | 0.00 | 0.00 | ± | 0.00 | 0.00 | ± | 0.00 | 0.00 | ± | 0.00 |  |
| Brown fat | 0.10 | ± | 0.00 | 0.08 | ± | 0.05 | 0.03 | ± | 0.00 | 0.02 | ± | 0.00 | 0.02 | ± | 0.00 | 0.01 | ± | 0.00 |  |
| Genital tract | 0.38 | ± | 0.16 | 0.17 | ± | 0.04 | 0.10 | ± | 0.03 | 0.07 | ± | 0.01 | 0.06 | ± | 0.04 | 0.05 | ± | 0.03 |  |
| Heart | 0.11 | ± | 0.02 | 0.05 | ± | 0.01 | 0.03 | ± | 0.00 | 0.01 | ± | 0.00 | 0.01 | ± | 0.00 | 0.01 | ± | 0.00 |  |
| Intestine | 0.60 | ± | 0.27 | 0.26 | ± | 0.09 | 0.04 | ± | 0.01 | 0.01 | ± | 0.00 | 0.01 | ± | 0.00 | 0.00 | ± | 0.00 |  |
| Kidney | 10.17 | ± | 1.01 | 9.05 | ± | 1.50 | 4.01 | ± | 0.52 | 2.16 | ± | 0.21 | 1.36 | ± | 0.14 | 0.45 | ± | 0.04 |  |
| Liver | 0.99 | ± | 0.11 | 0.57 | ± | 0.22 | 0.37 | ± | 0.04 | 0.29 | ± | 0.04 | 0.27 | ± | 0.02 | 0.24 | ± | 0.17 |  |
| Lung | 0.54 | ± | 0.10 | 0.25 | ± | 0.10 | 0.06 | ± | 0.02 | 0.05 | ± | 0.03 | 0.07 | ± | 0.05 | 0.02 | ± | 0.03 |  |
| Lymph nodes | 0.29 | ± | 0.04 | 0.15 | ± | 0.06 | 0.08 | ± | 0.04 | 0.06 | ± | 0.04 | 0.07 | ± | 0.02 | 0.08 | ± | 0.07 |  |
| Muscle | 0.05 | ± | 0.01 | 0.02 | ± | 0.00 | 0.01 | ± | 0.00 | 0.01 | ± | 0.00 | 0.00 | ± | 0.00 | 0.00 | ± | 0.00 |  |
| Ovaries | 0.23 | ± | 0.08 | 0.15 | ± | 0.03 | 0.06 | ± | 0.02 | 0.06 | ± | 0.02 | 0.06 | ± | 0.05 | 0.02 | ± | 0.02 |  |
| Pancreas | 0.13 | ± | 0.03 | 0.06 | ± | 0.01 | 0.03 | ± | 0.00 | 0.02 | ± | 0.00 | 0.01 | ± | 0.00 | 0.01 | ± | 0.01 |  |
| Salivary glands | 0.12 | ± | 0.00 | 0.07 | ± | 0.02 | 0.04 | ± | 0.01 | 0.03 | ± | 0.02 | 0.02 | ± | 0.00 | 0.01 | ± | 0.01 |  |
| Skin | 0.30 | ± | 0.06 | 0.18 | ± | 0.03 | 0.08 | ± | 0.02 | 0.05 | ± | 0.00 | 0.03 | ± | 0.00 | 0.02 | ± | 0.01 |  |
| Spleen | 0.60 | ± | 0.16 | 0.24 | ± | 0.12 | 0.27 | ± | 0.07 | 0.21 | ± | 0.03 | 0.18 | ± | 0.06 | 0.14 | ± | 0.03 |  |
| Stomach | 0.21 | ± | 0.06 | 0.07 | ± | 0.01 | 0.03 | ± | 0.00 | 0.02 | ± | 0.00 | 0.02 | ± | 0.00 | 0.01 | ± | 0.00 |  |
| **Tumor HCC70** | **2.27** | **±** | **0.40** | **0.70** | **±** | **0.15** | **0.21** | **±** | **0.01** | **0.14** | **±** | **0.01** | **0.09** | **±** | **0.02** | **0.04** | **±** | **0.01** |  |
| White fat | 0.11 | ± | 0.01 | 0.07 | ± | 0.01 | 0.03 | ± | 0.01 | 0.02 | ± | 0.01 | 0.01 | ± | 0.00 | 0.02 | ± | 0.02 |  |

**Table 7.** Tumor-to-organ ratios of %ID/g values of mice bearing HCC70 tumors injected with the **[^177^Lu]Lu-DOTA-A1K2** sdAb for the *in vivo* biodistribution study over time. Expressed as mean ± SD (standard deviation) (n=4)

|  | **2h p.i.** | | | **6h p.i.** | | | **24h p.i.** | | | **48h p.i.** | | | **72h p.i.** | | | **168h p.i.** | | | |
| --- | --- | --- | --- | --- | --- | --- | --- | --- | --- | --- | --- | --- | --- | --- | --- | --- | --- | --- | --- |
|  | Mean | ± | SD | Mean | ± | SD | Mean | ± | SD | Mean | ± | SD | Mean | ± | SD | Mean | ± | SD |  |
| Tumor/Blood | 13.41 | ± | 2.34 | 25.50 | ± | 7.80 | 30.09 | ± | 3.37 | 38.81 | ± | 4.99 | 30.33 | ± | 10.05 | 49.50 | ± | 6.38 |  |
| Tumor/Liver | 2.28 | ± | 0.26 | 1.35 | ± | 0.46 | 0.58 | ± | 0.04 | 0.48 | ± | 0.05 | 0.34 | ± | 0.08 | 0.20 | ± | 0.10 |  |
| Tumor/Muscle | 41.51 | ± | 2.96 | 31.85 | ± | 4.74 | 23.54 | ± | 1.19 | 22.16 | ± | 2.23 | 22.96 | ± | 7.19 | 8.73 | ± | 5.10 |  |
| Tumor/Kidney | 0.22 | ± | 0.03 | 0.08 | ± | 0.02 | 0.05 | ± | 0.00 | 0.06 | ± | 0.01 | 0.07 | ± | 0.02 | 0.08 | ± | 0.01 |  |

**Table 8.** *Ex vivo* biodistribution data obtained in mice bearing HCC70 tumors injected with the [**^161^Tb]Tb-DOTA-A1K2** sdAb, at different time points post-injection (p.i.). Expressed as mean ± SD (standard deviation) (n=4)

**Table 9.** Tumor-to-organ ratios of %ID/g values of mice bearing HCC70 tumors injected with the sdAb [**^161^Tb]Tb-DOTA-A1K2** for the *in vivo* biodistribution study over time. Expressed as mean ± SD (standard deviation) (n=4)

|  | **2h p.i.** | | | **6h p.i.** | | | **24h p.i.** | | | **48h p.i.** | | | **72h p.i.** | | | **168h p.i.** | | | |
| --- | --- | --- | --- | --- | --- | --- | --- | --- | --- | --- | --- | --- | --- | --- | --- | --- | --- | --- | --- |
| Organs | Mean | ± | SD | Mean | ± | SD | Mean | ± | SD | Mean | ± | SD | Mean | ± | SD | Mean | ± | SD |  |
| Blood | 0.07 | ± | 0.02 | 0.02 | ± | 0.00 | 0.01 | ± | 0.00 | 0.00 | ± | 0.00 | 0.00 | ± | 0.00 | 0.00 | ± | 0.00 |  |
| Bone | 0.12 | ± | 0.02 | 0.07 | ± | 0.01 | 0.08 | ± | 0.01 | 0.06 | ± | 0.01 | 0.05 | ± | 0.03 | 0.05 | ± | 0.01 |  |
| Brain | 0.01 | ± | 0.00 | 0.00 | ± | 0.00 | 0.00 | ± | 0.00 | 0.00 | ± | 0.00 | 0.00 | ± | 0.00 | 0.00 | ± | 0.00 |  |
| Brown fat | 0.06 | ± | 0.01 | 0.04 | ± | 0.01 | 0.02 | ± | 0.01 | 0.01 | ± | 0.00 | 0.01 | ± | 0.01 | 0.01 | ± | 0.01 |  |
| Genital tract | 0.22 | ± | 0.02 | 0.13 | ± | 0.03 | 0.07 | ± | 0.04 | 0.06 | ± | 0.02 | 0.03 | ± | 0.01 | 0.03 | ± | 0.02 |  |
| Heart | 0.08 | ± | 0.01 | 0.04 | ± | 0.00 | 0.02 | ± | 0.01 | 0.02 | ± | 0.01 | 0.01 | ± | 0.01 | 0.01 | ± | 0.00 |  |
| Intestine | 0.78 | ± | 0.28 | 0.57 | ± | 0.23 | 0.04 | ± | 0.00 | 0.05 | ± | 0.02 | 0.02 | ± | 0.01 | 0.01 | ± | 0.00 |  |
| Kidney | 7.59 | ± | 1.07 | 6.10 | ± | 0.53 | 2.90 | ± | 1.00 | 1.71 | ± | 0.46 | 1.29 | ± | 0.24 | 0.47 | ± | 0.15 |  |
| Liver | 0.25 | ± | 0.03 | 0.19 | ± | 0.04 | 0.13 | ± | 0.05 | 0.09 | ± | 0.03 | 0.08 | ± | 0.01 | 0.04 | ± | 0.01 |  |
| Lung | 0.41 | ± | 0.05 | 0.18 | ± | 0.02 | 0.06 | ± | 0.00 | 0.05 | ± | 0.05 | 0.03 | ± | 0.00 | 0.01 | ± | 0.00 |  |
| Lymph nodes | 0.21 | ± | 0.12 | 0.12 | ± | 0.01 | 0.08 | ± | 0.05 | 0.09 | ± | 0.01 | 0.07 | ± | 0.02 | 0.02 | ± | 0.02 |  |
| Muscle | 0.04 | ± | 0.01 | 0.03 | ± | 0.00 | 0.02 | ± | 0.01 | 0.01 | ± | 0.01 | 0.01 | ± | 0.00 | 0.00 | ± | 0.00 |  |
| Ovaries | 0.17 | ± | 0.03 | 0.09 | ± | 0.01 | 0.07 | ± | 0.03 | 0.05 | ± | 0.03 | 0.04 | ± | 0.01 | 0.02 | ± | 0.01 |  |
| Pancreas | 0.11 | ± | 0.01 | 0.05 | ± | 0.00 | 0.02 | ± | 0.01 | 0.02 | ± | 0.00 | 0.02 | ± | 0.01 | 0.01 | ± | 0.00 |  |
| Salivary glands | 0.09 | ± | 0.02 | 0.06 | ± | 0.01 | 0.04 | ± | 0.02 | 0.02 | ± | 0.01 | 0.02 | ± | 0.01 | 0.01 | ± | 0.00 |  |
| Skin | 0.26 | ± | 0.11 | 0.20 | ± | 0.09 | 0.11 | ± | 0.04 | 0.06 | ± | 0.01 | 0.04 | ± | 0.02 | 0.02 | ± | 0.01 |  |
| Spleen | 0.18 | ± | 0.04 | 0.11 | ± | 0.04 | 0.09 | ± | 0.05 | 0.08 | ± | 0.03 | 0.06 | ± | 0.04 | 0.03 | ± | 0.01 |  |
| Stomach | 0.39 | ± | 0.26 | 0.13 | ± | 0.08 | 0.04 | ± | 0.01 | 0.07 | ± | 0.04 | 0.02 | ± | 0.01 | 0.01 | ± | 0.00 |  |
| **Tumor HCC70** | **1.03** | **±** | **0.17** | **0.46** | **±** | **0.04** | **0.24** | **±** | **0.07** | **0.11** | **±** | **0.04** | **0.10** | **±** | **0.01** | **0.04** | **±** | **0.01** |  |
| White fat | 0.09 | ± | 0.03 | 0.04 | ± | 0.00 | 0.02 | ± | 0.01 | 0.01 | ± | 0.00 | 0.02 | ± | 0.03 | 0.01 | ± | 0.00 |  |

|  | **2h p.i.** | | | **6h p.i.** | | | **24h p.i.** | | | **48h p.i.** | | | **72h p.i.** | | | **168h p.i.** | | | |
| --- | --- | --- | --- | --- | --- | --- | --- | --- | --- | --- | --- | --- | --- | --- | --- | --- | --- | --- | --- |
|  | Mean | ± | SD | Mean | ± | SD | Mean | ± | SD | Mean | ± | SD | Mean | ± | SD | Mean | ± | SD |  |
| Tumor/Blood | 16.95 | ± | 8.78 | 30.50 | ± | 11.26 | 44.76 | ± | 26.73 | 37.94 | ± | 11.25 | 42.27 | ± | 31.95 | 106.43 | ± | 60.03 |  |
| Tumor/Liver | 4.22 | ± | 0.85 | 2.44 | ± | 0.50 | 2.09 | ± | 0.84 | 1.29 | ± | 0.61 | 1.24 | ± | 0.24 | 1.04 | ± | 0.34 |  |
| Tumor/Muscle | 25.21 | ± | 8.40 | 18.35 | ± | 5.24 | 13.41 | ± | 3.21 | 13.03 | ± | 6.96 | 18.56 | ± | 4.59 | 15.27 | ± | 10.40 |  |
| Tumor/Kidney | 0.14 | ± | 0.03 | 0.08 | ± | 0.00 | 0.09 | ± | 0.04 | 0.06 | ± | 0.02 | 14.93 | ± | 29.70 | 0.09 | ± | 0.03 |  |

|  | **2h p.i.** | | | **6h p.i.** | | | **24h p.i.** | | | **48h p.i.** | | | **72h p.i.** | | | **168h p.i.** | | | |
| --- | --- | --- | --- | --- | --- | --- | --- | --- | --- | --- | --- | --- | --- | --- | --- | --- | --- | --- | --- |
| Organs | Mean | ± | SD | Mean | ± | SD | Mean | ± | SD | Mean | ± | SD | Mean | ± | SD | Mean | ± | SD |  |
| Blood | 0.09 | ± | 0.03 | 0.04 | ± | 0.02 | 0.01 | ± | 0.00 | 0.00 | ± | 0.00 | 0.00 | ± | 0.00 | / |  |  |  |
| Bone | 0.28 | ± | 0.15 | 0.19 | ± | 0.06 | 0.24 | ± | 0.04 | 0.14 | ± | 0.05 | 0.23 | ± | 0.16 | 0.20 | ± | 0.03 |  |
| Brain | 0.01 | ± | 0.00 | 0.00 | ± | 0.00 | 0.00 | ± | 0.00 | 0.00 | ± | 0.00 | 0.00 | ± | 0.00 | 0.00 | ± | 0.00 |  |
| Brown fat | 0.08 | ± | 0.02 | 0.06 | ± | 0.01 | 0.03 | ± | 0.01 | 0.01 | ± | 0.00 | 0.02 | ± | 0.00 | 0.01 | ± | 0.00 |  |
| Genital tract | 0.23 | ± | 0.04 | 0.18 | ± | 0.04 | 0.11 | ± | 0.05 | 0.05 | ± | 0.01 | 0.08 | ± | 0.06 | 0.04 | ± | 0.03 |  |
| Heart | 0.10 | ± | 0.04 | 0.06 | ± | 0.00 | 0.03 | ± | 0.01 | 0.01 | ± | 0.00 | 0.02 | ± | 0.00 | 0.01 | ± | 0.00 |  |
| Intestine | 0.77 | ± | 0.35 | 0.90 | ± | 0.20 | 0.07 | ± | 0.03 | 0.03 | ± | 0.01 | 0.01 | ± | 0.00 | 0.01 | ± | 0.00 |  |
| Kidney | **7.82** | **±** | **0.36** | **8.14** | **±** | **0.95** | **4.44** | **±** | **0.97** | **2.37** | **±** | **0.67** | **1.89** | **±** | **0.14** | **0.74** | **±** | **0.11** |  |
| Liver | 1.01 | ± | 0.40 | 0.56 | ± | 0.19 | 0.39 | ± | 0.17 | 0.21 | ± | 0.10 | 0.25 | ± | 0.08 | 0.15 | ± | 0.03 |  |
| Lung | 0.52 | ± | 0.18 | 0.22 | ± | 0.06 | 0.07 | ± | 0.00 | 0.07 | ± | 0.06 | 0.10 | ± | 0.03 | 0.16 | ± | 0.24 |  |
| Lymph nodes | 0.21 | ± | 0.08 | 0.17 | ± | 0.06 | 0.11 | ± | 0.02 | 0.05 | ± | 0.03 | 0.13 | ± | 0.07 | 0.06 | ± | 0.05 |  |
| Muscle | 0.04 | ± | 0.00 | 0.05 | ± | 0.04 | 0.01 | ± | 0.01 | 0.01 | ± | 0.01 | 0.07 | ± | 0.11 | 0.01 | ± | 0.01 |  |
| Ovaries | 0.20 | ± | 0.02 | 0.14 | ± | 0.04 | 0.09 | ± | 0.03 | 0.04 | ± | 0.01 | 0.10 | ± | 0.06 | 0.03 | ± | 0.01 |  |
| Pancreas | 0.11 | ± | 0.02 | 0.06 | ± | 0.01 | 0.03 | ± | 0.01 | 0.01 | ± | 0.00 | 0.02 | ± | 0.00 | 0.01 | ± | 0.00 |  |
| Salivary glands | 0.09 | ± | 0.01 | 0.06 | ± | 0.01 | 0.03 | ± | 0.00 | 0.02 | ± | 0.01 | 0.02 | ± | 0.00 | 0.01 | ± | 0.00 |  |
| Skin | 0.21 | ± | 0.05 | 0.15 | ± | 0.02 | 0.09 | ± | 0.04 | 0.04 | ± | 0.02 | 0.05 | ± | 0.03 | 0.03 | ± | 0.02 |  |
| Spleen | 0.25 | ± | 0.05 | 0.13 | ± | 0.02 | 0.14 | ± | 0.02 | 0.07 | ± | 0.03 | 0.22 | ± | 0.07 | 0.20 | ± | 0.12 |  |
| Stomach | 0.23 | ± | 0.09 | 0.17 | ± | 0.10 | 0.04 | ± | 0.02 | 0.02 | ± | 0.00 | 0.02 | ± | 0.00 | 0.01 | ± | 0.00 |  |
| Tumor MDA-MB-231 hMSLN+ | **21.88** | **±** | **5.47** | **16.34** | **±** | **10.77** | **5.85** | **±** | **4.73** | **3.68** | **±** | **3.78** | **3.64** | **±** | **1.52** | **1.68** | **±** | **0.65** |  |
| Tumor MDA-MB-231 hMSLN- | **0.49** | **±** | **0.11** | **0.27** | **±** | **0.01** | **0.15** | **±** | **0.02** | **0.07** | **±** | **0.02** | **0.13** | **±** | **0.01** | **0.05** | **±** | **0.01** |  |
| White fat | 0.11 | ± | 0.01 | 0.09 | ± | 0.03 | 0.03 | ± | 0.01 | 0.01 | ± | 0.00 | 0.01 | ± | 0.00 | 0.01 | ± | 0.00 |  |

**Table 10.** *Ex vivo* biodistribution data obtained in mice bearing MDA-MB-231 hMSLN+ or hMSLN- tumors injected with the [**^161^Tb]Tb-DOTA-A1K2** sdAb, at different time points post-injection (p.i.). Expressed as mean ± SD (standard deviation) (n=4)

**Table 11.** Tumor-to-organ ratios of %ID/g values of mice bearing MDA-MB-231 hMSLN+ or hMSLN- tumors injected with the [**^161^Tb]Tb-DOTA-A1K2** sdAb for the *in vivo* biodistribution study over time. Expressed as mean ± SD (standard deviation) (n=4)

|  |  | **2h p.i.** | | | **6h p.i.** | | | **24h p.i.** | | | **48h p.i.** | | | **72h p.i.** | | | | **168h p.i.** | | |
| --- | --- | --- | --- | --- | --- | --- | --- | --- | --- | --- | --- | --- | --- | --- | --- | --- | --- | --- | --- | --- |
|  |  | Mean | ± | SD | Mean | ± | SD | Mean | ± | SD | Mean | ± | SD | Mean | ± | SD | Mean | | ± | SD |
| Tumor MDA-MB-231 hMSLN+ | Tumor/Blood | 267.45 | ± | 83.64 | 416.54 | ± | 78.63 | 890.18 | ± | 395.09 | 1492.77 | ± | 1654.56 | 1887.62 | ± | 527.58 | / | |  |  |
|  | Tumor/Liver | 23.33 | ± | 7.17 | 28.19 | ± | 12.39 | 13.44 | ± | 4.40 | 16.51 | ± | 13.35 | 15.15 | ± | 5.73 | 10.91 | | ± | 3.50 |
|  | Tumor/Muscle | 553.98 | ± | 123.07 | 418.22 | ± | 240.35 | 475.64 | ± | 408.08 | 383.80 | ± | 120.13 | 330.23 | ± | 262.64 | 386.87 | | ± | 214.68 |
|  | Tumor/Kidney | 2.79 | ± | 0.65 | 2.00 | ± | 1.33 | 1.25 | ± | 0.76 | 1.43 | ± | 1.24 | 1.92 | ± | 0.74 | 2.32 | | ± | 0.98 |
| Tumor MDA-MB-231 hMSLN- | Tumor/Blood | 5.84 | ± | 1.05 | 8.63 | ± | 4.29 | 26.20 | ± | 5.00 | 29.06 | ± | 8.34 | 70.46 | ± | 15.29 | / | |  |  |
|  | Tumor/Liver | 0.51 | ± | 0.11 | 0.53 | ± | 0.22 | 0.42 | ± | 0.12 | 0.38 | ± | 0.08 | 0.57 | ± | 0.20 | 0.36 | | ± | 0.04 |
|  | Tumor/Muscle | 12.42 | ± | 2.72 | 8.96 | ± | 7.43 | 12.04 | ± | 4.44 | 10.74 | ± | 4.12 | 10.55 | ± | 6.77 | 13.55 | | ± | 7.05 |
|  | Tumor/Kidney | 0.06 | ± | 0.01 | 0.03 | ± | 0.00 | 0.03 | ± | 0.00 | 0.03 | ± | 0.00 | 0.07 | ± | 0.01 | 0.08 | | ± | 0.02 |

**
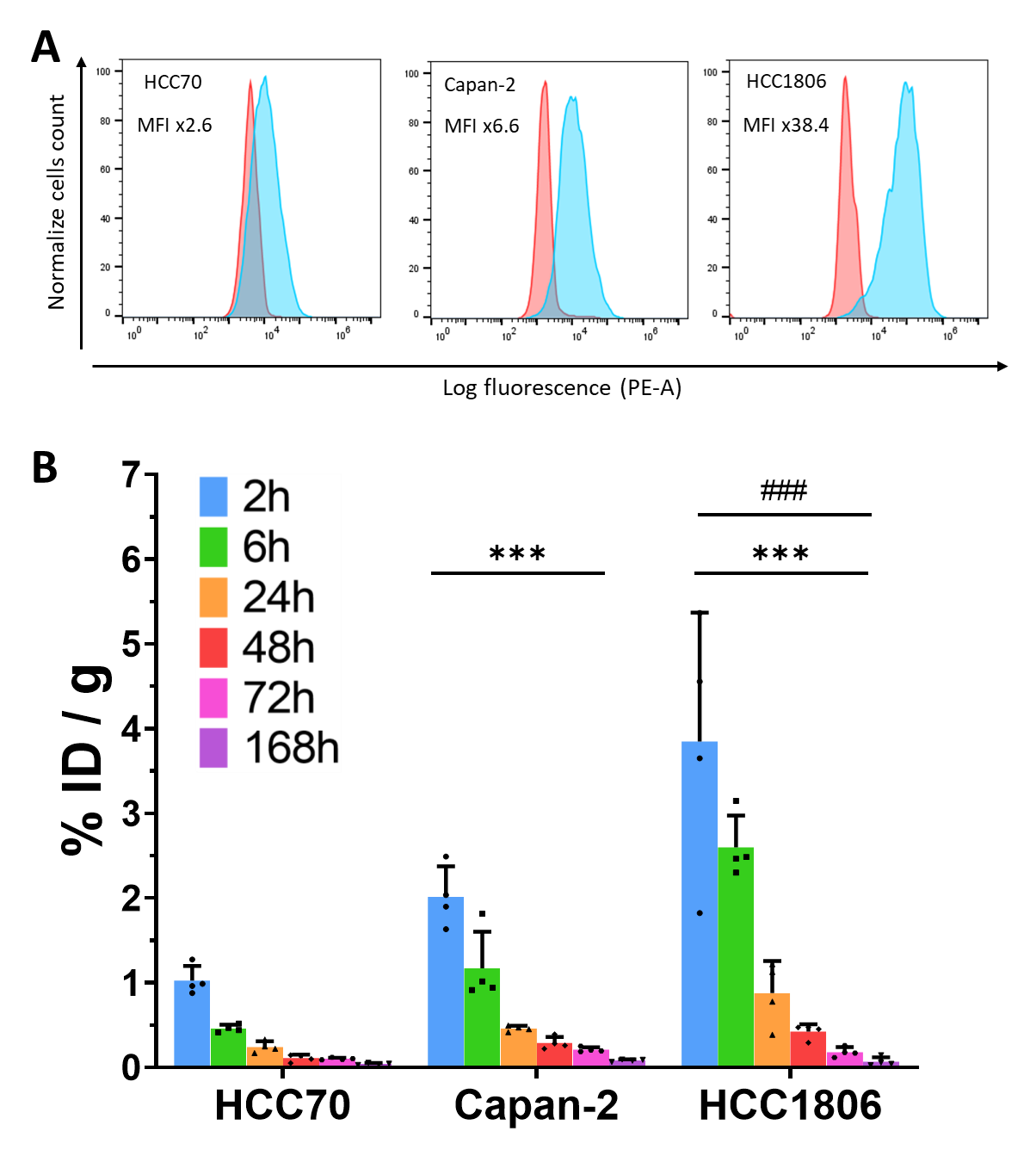
**

**SFig 1. | MSLN expression and** [**^161^Tb]Tb-DOTA-A1K2** **uptake in three different tumor types**

1. MSLN expression determined in HCC70, Capan-2 and HCC1806 cell lines by FACS analysis. MFI =Median Fluorescence Intensity
2. Tumor uptake determined from 2 to 168 hours after i.v. co-injection of [^161^Tb]Tb-DOTA-A1K2 and Gelofusin, in mice bearing HCC70, Capan-2 or HCC1806 xenografts. (n=4). *Significantly different from HCC70 uptake (***p < 0.001). ^#^Significantly different from Capan-2 uptake (^###^p < 0.001).


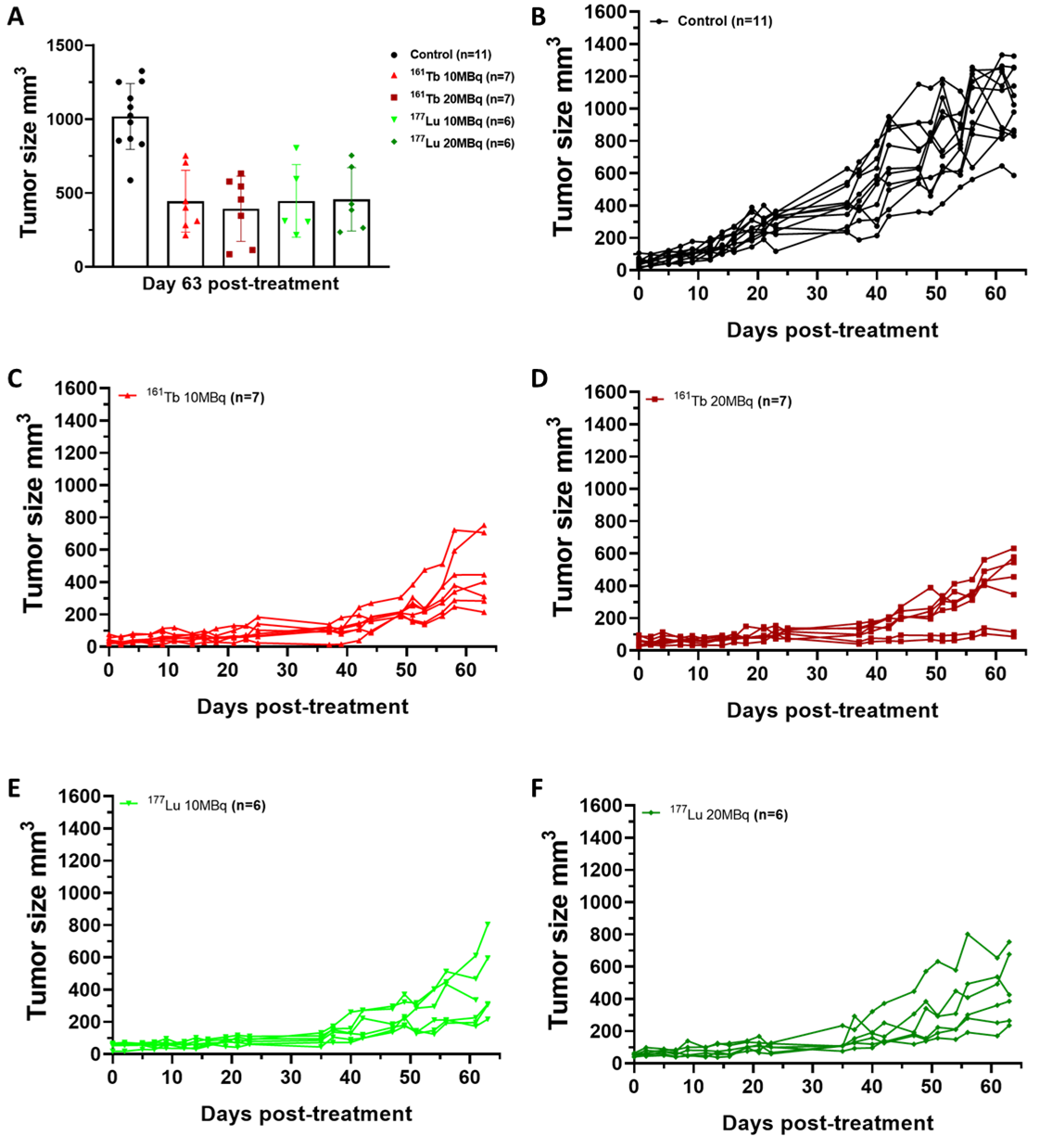
**SFig 2. | Graphs representing individual MDA-MB-231 MSLN+ tumor growth of mice in the tumor therapy study with the higher injected activities.**

1. Tumor size in mm^3^ the last day of the experiment, 63 days post- injection
2. Individual mice treated with saline solution (control), n=11
3. Individual mice treated with 10 MBq of [^161^Tb]Tb-DOTA-A1K2, n=7
4. Individual mice treated with 20 MBq of [^161^Tb]Tb-DOTA-A1K2, n=7
5. Individual mice treated with 10 MBq of [^177^Lu]Lu-DOTA-A1K2, n=6
6. Individual mice treated with 20 MBq of [^177^Lu]Lu-DOTA-A1K2, n=6


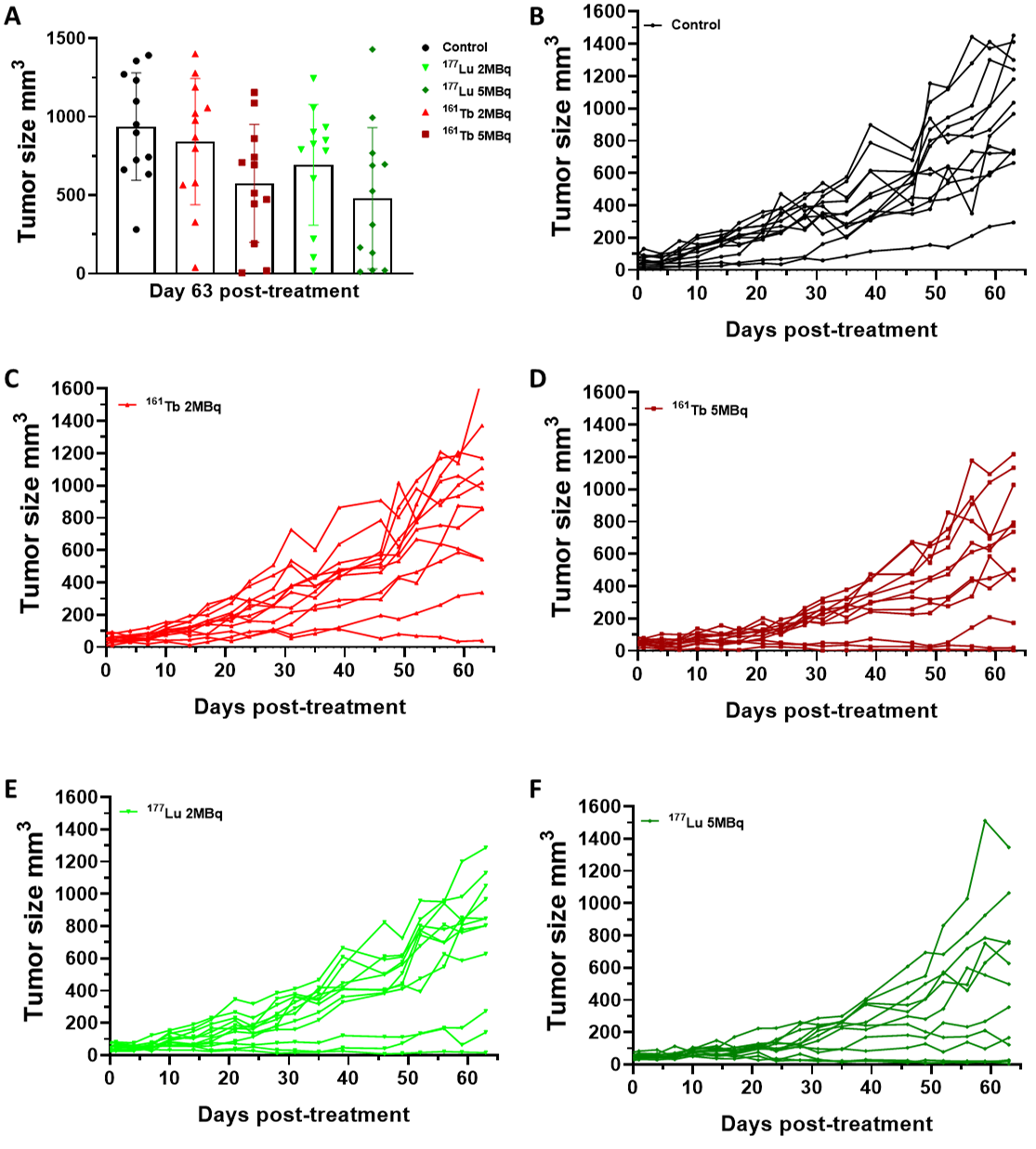


**SFig 3. | Graphs representing individual MDA-MB-231 MSLN+ tumor growth of mice in the tumor therapy study with the lower injected activities.**

1. Tumor size in mm^3^ the last day of the experiment, 63 days post- injection
2. Individual mice treated with saline solution (control), n=12
3. Individual mice treated with 2 MBq of [^161^Tb]Tb-DOTA-A1K2, n=12
4. Individual mice treated with 5 MBq of [^161^Tb]Tb-DOTA-A1K2, n=12
5. Individual mice treated with 2 MBq of [^177^Lu]Lu-DOTA-A1K2, n=12
6. Individual mice treated with 5 MBq of [^177^Lu]Lu-DOTA-A1K2, n=12


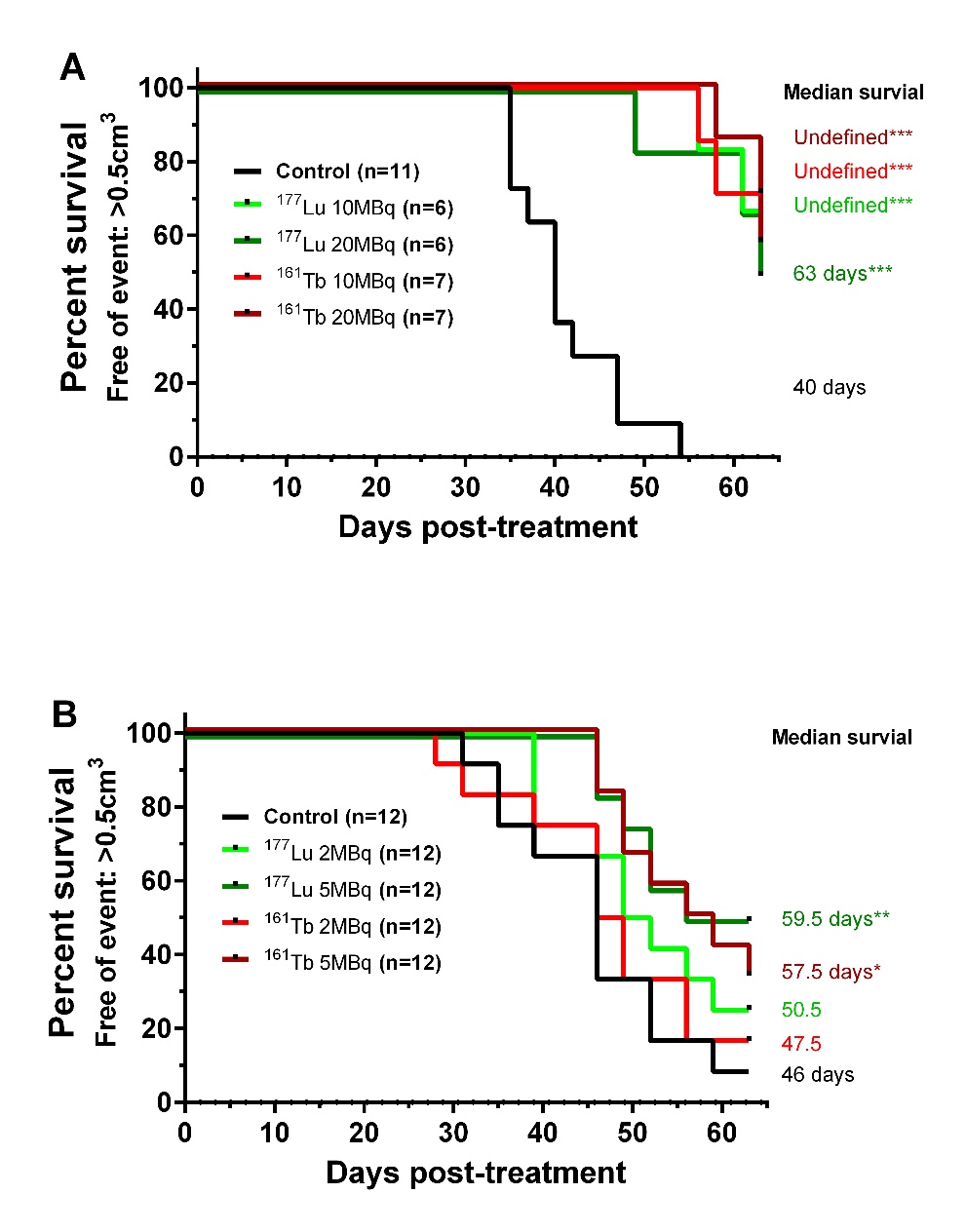


**SFig 4. | survival of mice, free of the event: tumor >500mm^3^**

1. Kaplan-Meier plot with survival curves of control mice in the tumor therapy study with the higher injected activities. ***p < 0.001 *vs* control group.
2. Kaplan-Meier plot with survival curves of control mice in the tumor therapy study with the lower injected activities. **p < 0.01 and *p < 0.05 vs control group.

**
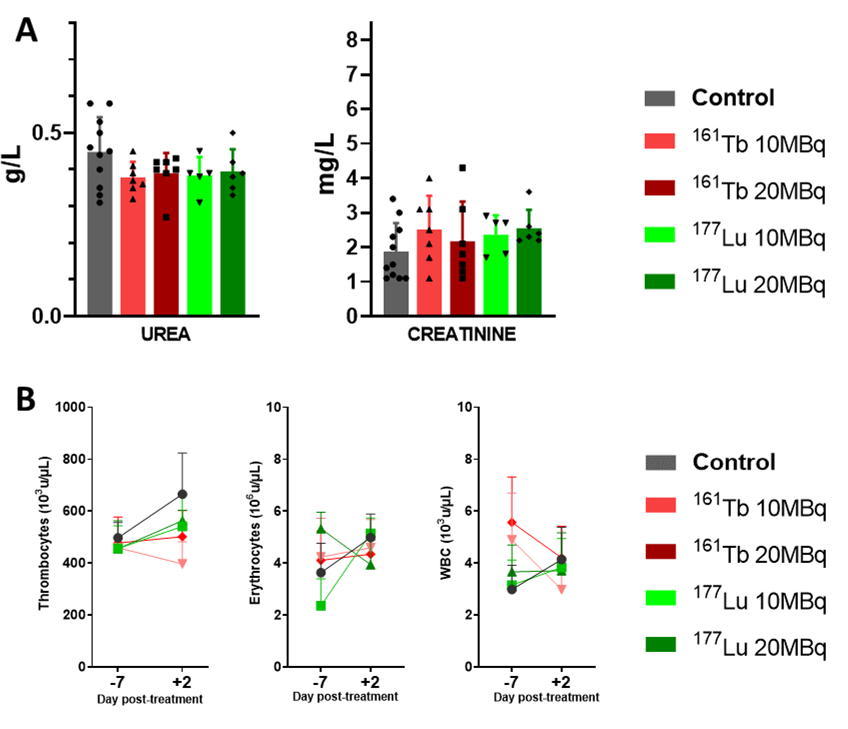
SFig 5. | Graphs representing the *in vivo* toxicity study from the mice treated with the higher injected activities.**

1. Blood plasma biomarkers of kidneys function (urea and creatine) quantified using samples obtained on the day of euthanasia. Values did not significantly differ from each other.
2. Thrombocytes, erythrocytes and leukocytes count 7 days before and 2 days after treatment. No statistical differences were observed.

**
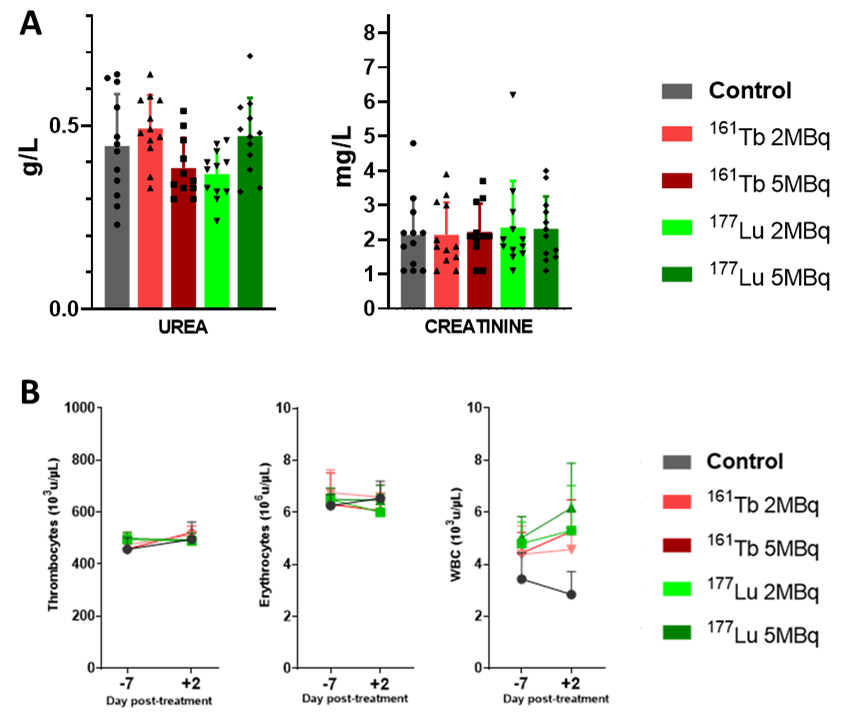
**

**SFig 6. | Graphs representing the *in vivo* toxicity study from the mice treated with the lower injected activities.**

1. Blood plasma biomarkers of kidneys function (urea and creatine) quantified using samples obtained on the day of euthanasia. Values did not significantly differ from each other.
2. Thrombocytes, erythrocytes and leukocytes count 7 days before and 2 days after treatment. No statistical differences were observed.
